# Iron Oxide Nanoparticles in Regenerative Medicine and Tissue Engineering

**DOI:** 10.3390/nano11092337

**Published:** 2021-09-08

**Authors:** Ralf P. Friedrich, Iwona Cicha, Christoph Alexiou

**Affiliations:** Department of Otorhinolaryngology, Head and Neck Surgery, Section of Experimental Oncology and Nanomedicine (SEON), Else Kröner-Fresenius-Stiftung Professorship, Universitätsklinikum Erlangen, 91054 Erlangen, Germany; Iwona.Cicha@uk-erlangen.de (I.C.); christoph.alexiou@uk-erlangen.de (C.A.)

**Keywords:** superparamagnetic iron oxide nanoparticles, SPION, magnetic drug delivery, magnetic resonance imaging, magnetic particles, nanomedicine

## Abstract

In recent years, many promising nanotechnological approaches to biomedical research have been developed in order to increase implementation of regenerative medicine and tissue engineering in clinical practice. In the meantime, the use of nanomaterials for the regeneration of diseased or injured tissues is considered advantageous in most areas of medicine. In particular, for the treatment of cardiovascular, osteochondral and neurological defects, but also for the recovery of functions of other organs such as kidney, liver, pancreas, bladder, urethra and for wound healing, nanomaterials are increasingly being developed that serve as scaffolds, mimic the extracellular matrix and promote adhesion or differentiation of cells. This review focuses on the latest developments in regenerative medicine, in which iron oxide nanoparticles (IONPs) play a crucial role for tissue engineering and cell therapy. IONPs are not only enabling the use of non-invasive observation methods to monitor the therapy, but can also accelerate and enhance regeneration, either thanks to their inherent magnetic properties or by functionalization with bioactive or therapeutic compounds, such as drugs, enzymes and growth factors. In addition, the presence of magnetic fields can direct IONP-labeled cells specifically to the site of action or induce cell differentiation into a specific cell type through mechanotransduction.

## 1. Introduction

The number of publications related to the field of regenerative medicine have increased dramatically over the last 20 years. Nanotechnology is also finding its way into biomedical research and could significantly improve and accelerate tissue regeneration due to its versatility and functionalization possibilities. The materials used are composed of a wide variety of components and exhibit a broad range of shapes, from fibrous structures or nanopatterned surfaces to particulate objects. Particulate materials, such as NPs, are specifically utilized for diagnostic purposes, as they enable multimodal and multifunctional molecular imaging [1,2]. Moreover, they can easily be modified to serve as vehicles for the transport of drugs or genes and they are also increasingly used in cell therapy and tissue engineering approaches [3,4,5]. Especially in stem cell therapy, they can be applied to trigger a desired cell differentiation by positively influencing regeneration per se, coupled with bioactive substances and/or in the presence of magnetic fields. Similarly, they can be used in tissue engineering approaches as part of a biocompatible scaffold in combination with cells and/or bioactive substances to support the regeneration or replacement of damaged cells or tissue. Despite the stringent requirements for biocompatibility, scaffolds made of different materials with a wide variety of surface properties and structures have been developed to create conditions for cell adhesion and proliferation that are optimized for the particular applications.

One of the most promising nanoscale materials are iron oxide nanoparticles (IONPs), which can be functionalized with other bioactive substances, embedded in composites, and bound or taken up by cells [6]. IONPs are frequently used for drug delivery applications, where a directed drug delivery is enabled either by the use of specific binding proteins, such as antibodies, or by the influence of external magnetic fields [7,8]. Furthermore, primary cells and cell lines can be magnetically labeled with IONPs, allowing non-invasive in vivo monitoring of the efficacy of a cell therapy or tissue engineering approaches using magnetic resonance imaging (MRI) if the particle loading is sufficient [9,10]. In this context, it is worth noting that cells with high phagocytic activity, e.g., macrophages, enable the uptake of high amounts of NPs without the need for transfection agents, whereas other cells, such as T cells or stem cells, may require transfection agents, peptide-based delivery systems or magnetotransfection to achieve an adequate IONP loading [11,12,13]. In any case, IONPs can provide information about the correct positioning and function of cells and materials over time and allow monitoring of the degradation or clearance of scaffold materials. Moreover, IONPs-labelled cells can be magnetically directed to a specific location to enhance the regeneration of a tissue or to restore a function [14]. Since stem cells are also capable of differentiating into a variety of other cells, including myoblasts, adipocytes, chondrocytes, osteoblasts and neuron-like cells, the applications of IONPs-loaded cells in regenerative medicine are immense. Finally, biomaterials and particle-loaded cells can be used to create biocompatible structures that can be implanted as grafts to replace destroyed or diseased tissue. Even without the use of artificial support structures, it is possible to generate three-dimensional tissue such as cell sheets and spheroids using particle-loaded cells [15].

The successful use of NP-based cell therapy and tissue engineering and its monitoring by MRI for regeneration or replacement of diseased or injured tissue has been demonstrated in numerous preclinical in vitro and in vivo studies. This review highlights developments in regenerative medicine and focuses on the use of iron oxide nanoparticles in cell therapy and tissue engineering. Outlined is not only the use of IONPs for cell tracking by MRI, but in particular the active use of these particles to promote cell and tissue regeneration in cardiovascular, osteochondral and neuronal diseases and defects, among others. Excluded are the broad applications of IONPs in tumor treatment or imaging, as these have been described in detail elsewhere.

## 2. IONP Synthesis, Functionalization and Targeting

**Figure 1 nanomaterials-11-02337-f001:**
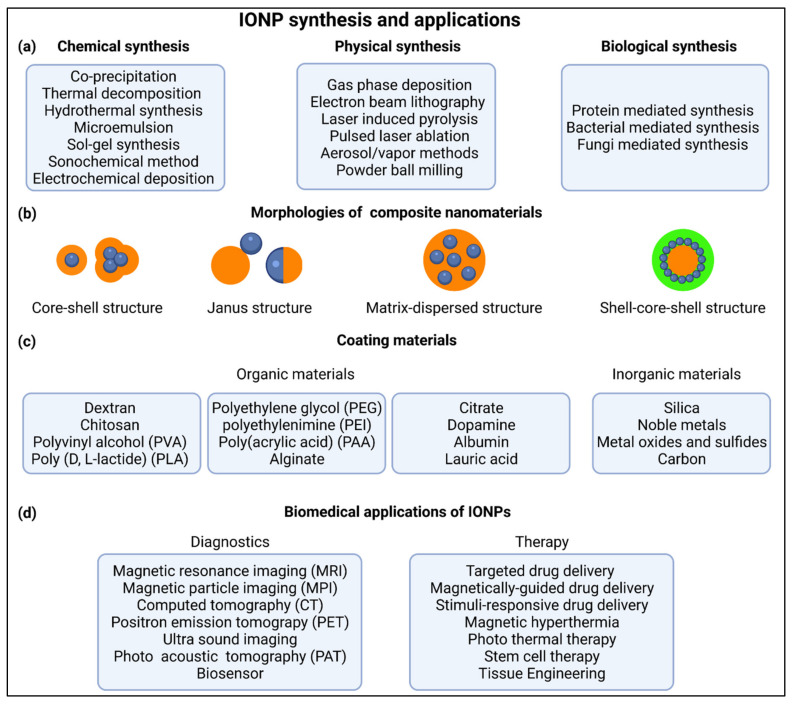
IONP synthesis and applications. (**a**) Common methods for chemical, physical and biologically based synthesis of IONPs. (**b**) Basic morphologies of iron oxide-based NPs (blue: iron oxide core; orange and green: coating materials). (**c**) Frequently used organic and inorganic materials for coating NPs. (**d**) IONP-mediated therapeutic and diagnostic procedures commonly used in biomedical research (Created with BioRender.com).

Over the past decades, there have been countless different IONPs developed for biomedical purposes. However, there are basically only a few synthesis methods used for the production of the raw particles, including physical, chemical or biosynthetic methods (Figure 1a) [11,16,17,18,19,20]. While physical and biosynthetic methods are utilized for the production of less than 10% of all IONPs, chemical methods, most notably coprecipitation, microemulsion and hydrothermal synthesis, account for the majority of reported production methods [16,17,21]. Since the initial synthesis determines to a large extent the basic properties of the particles, such as crystal structure, magnetizability, size, size distribution and shape, it must be carefully selected to best match the desired IONP properties [16,22].

The versatility and broad applicability of IONPs is achieved by surface coating and functionalization. This provides a certain stability and biocompatibility, which are crucial for many applications, and may equip the nanoparticle surface with targeted substances to ensure specific interaction, e.g., with cells. There are many strategies to increase the stability and achieve the necessary hydrophilicity of NPs for medical research. IONPs can be stabilized as part of a composite in core-shell structures, shell-core-shell structures, matrix-dispersed structures, Janus-type structures and combinations thereof (Figure 1b) [16]. However, it should be noted that the iron oxide core may exist as a single-core or as a multi-core particle [23].

In medical research, organic materials are the most frequently used surface coatings to prevent aggregation and increase stability and biocompatibility, and are applied either by an in-situ reaction or after the actual synthesis [16]. The available materials are diverse and often consist of polymers such as dextran, chitosan, polyethylene glycol (PEG), polyvinyl alcohol (PVA), etc. (Figure 1c) [11,24,25,26]. Apart from polymers, single small molecules such as citrate, lauric acid or proteins such as albumins are also widely utilized [6,11,27]. Besides organic compounds, inorganic materials, such as silica, carbon, noble metals, metal oxides and metal sulfides, have often been used as surface coatings to enhance optical absorption, electron density (e.g., Ag and Au) and magnetic moment (e.g., manganese or cobalt oxide) or to introduce features such as phosphorescence by doping with Y_2_O_3_ [16]. In addition, other bioactive molecules such as growth factors, enzymes, genes, drugs, antibodies or other substances containing a specific binding motif can be attached to existing reactive groups (e.g., –COOH, –OH, –NH_2_, –SH) of the primary coating or to linkers, enabling a more specific functionalization and targeting ability [16,18,24,28,29].

The functionalization of NPs enables their utilization in a variety of biotechnological and biomedical applications. They are used as biosensors, in diagnostics as contrast agent for magnetic resonance imaging (MRI), magnetic particle imaging (MPI), ultra sound (US), positron emission tomography (PET), photo acoustic tomography (PAT) and computed tomography (CT), and in the treatment of diseases, through targeted or stimuli-responsive drug delivery of bioactive agents, in tissue engineering and regeneration [28,30,31,32,33,34,35]. Due to their magnetic properties, applications such as hyperthermia or magnetically guided drug delivery are possible [26,34,36,37]. In addition, IONPs are suitable for theranostic purposes, such as image-guided and microbubble-mediated blood-brain barrier opening, image-guided drug delivery and theranostic tissue engineering [11,37].

The broad range of applications and the targeting specificity of IONPs are determined by both their physicochemical and biological particle properties. An important factor is the particle size, which is highly responsible for biodistribution and determines the ability to overcome biological barriers [29]. Generally, very small particles with a hydrodynamic diameter up to 5–8 nm are excreted by the kidneys. Larger NPs are easily captured by the reticuloendothelial system (RES), e.g., in the liver and spleen, or by macrophages, allowing liver and spleen imaging, as well as detection of inflammatory areas [11]. NPs in the size of about 20 nm to 150 nm may also accumulate in the stomach, bone, and kidney. However, medium-size particles are well suited for cancer imaging as they can be passively incorporated into tumors through the enhanced permeability and retention (EPR) effect. In addition, they can be used for drug delivery due to the relatively long circulating time in blood vessels. Coating with polymers, e.g., PEG, can significantly reduce opsonization with serum proteins and reduce removal by the RES, especially if a neutral surface charge is achieved [24,29]. In contrast, negatively or positively charged NPs are rapidly removed from the circulation by macrophages [29].

## 3. Cardiovascular Tissue Regeneration and Engineering

**Figure 2 nanomaterials-11-02337-f002:**
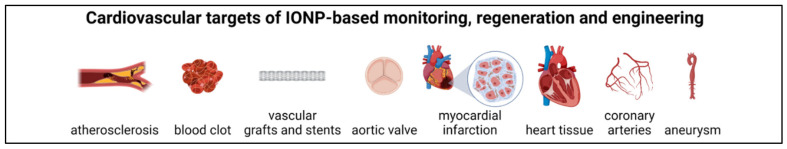
Possible targets for IONP-assisted cardiovascular tissue engineering and regeneration (Created with BioRender.com).

Over the decades, significant—albeit mainly experimental—progress has been made in the development of materials and substances for cardiovascular regeneration, including the functional restoration or production of e.g., blood vessels, heart valves and myocardium (Figure 2) [38]. Nanotechnology is playing an increasingly important role in the development of alternative cardiovascular therapies [39]. In addition, the design of nanoparticle probes for noninvasive imaging of cardiovascular targets, such as vascular inflammation, plaques, thrombosis, myocardial apoptosis, and angiogenesis, plays an increasingly important role in diagnostics and therapy monitoring [40,41,42,43,44,45]. In particular IONPs have decisively advanced cardiovascular research and experimental therapies, by enabling multimodal imaging and thus the diagnosis of diseases and the monitoring of pathological processes and therapy, but also by serving as carriers of therapeutic agents [46,47,48].

### 3.1. Thrombolysis

Thrombosis is a common condition with a significant mortality rate by triggering conditions such as stroke or myocardial infarction. Thus, drugs, such as tissue plasminogen activator (tPA), which catalyzes the conversion of plasminogen to plasmin and thereby dissolves clots, are used to treat ischemic stroke [49]. However, systemic treatment requires a relatively high dose, which can lead to severe side effects. Therefore, efforts are underway to develop techniques in which the thrombolytic drugs can exert their effects in a more targeted manner [50,51,52].

In a comparative study from our group, Friedrich et al. investigated different coupling methods to efficiently and functionally bind tPA to poly(acrylic acid-co-maleic acid)-coated IONPs [53]. We found that the loading efficiency, enzymatic activity and long-term stability of covalently coupled tPA were significantly higher than IONPs with adsorptively coupled tPA. In another study from the same group, tPA was bound to dextran-stabilized IONPs by carbodiimide-mediated amide bond formation and its functionality was confirmed on thrombus-mimicking fibrin-containing agarose gels as well as on blood plasma clots [54]. IONPs with covalently bound tPA were also used in studies by Chen et al. [55,56,57,58]. Using tPA covalently bound to carboxymethyldextran-coated IONPs, the authors detected no cytotoxicity and were able to demonstrate full retention of the thrombolytic activity of tPA [57]. Chitosan-coated and tPA-functionalized IONPs were found to be suitable for magnetically guided thrombolysis, reducing blood clot lysis time by 50% and 53%, respectively, compared with tests without magnetic targeting and free tPA [55]. Moreover, effective thrombolysis under magnetic guidance was also demonstrated in a rat embolism model, in which one-fifth the dose of tPA was able to produce comparable thrombolytic efficacy of the free drug. Similar results were obtained in a later study with silica-based IONPs [56]. In addition to good in vitro and in vivo biocompatibility, the SiO_2_-IONP-tPA particles also showed increased blood and storage stability. Furthermore, magnetically targeted thrombolysis in an ex vivo model resulted in a 34% reduction in blood clot lysis time compared to the required lysis time without magnetic targeting and 40% reduction compared to free tPA. In a further study, authors from the same group evaluated the possibility of a dual targeting strategy using poly(lactic-co-glycolic acid) (PLGA) IONPs functionalized with tPA and fibrin [59]. The particles showed complete retention of tPA activity, high biocompatibility, fibrin-binding effects and the possibility of magnetic guidance. Targeted in vivo thrombolysis was confirmed in a rat embolism model, in which tPA-IONPs at only one-fifth of the tPA dosage compared with free tPA were sufficient to restore blood flow in a vascular thrombus. The feasibility of magnetic targeting was also demonstrated in other studies. For example, after intravenous injection, a large local aggregation of tPA-IONPs was detected in the left iliac arteries of rats when a magnet was previously placed at appropriate sites [60]. Yang et al. developed poly [aniline-co-N-(1-one-butyric acid) aniline] coated IONPs functionalized with tPA [61]. The immobilized tPA not only showed improved storage stability and in vitro thrombolytic activity but also rapidly restored blood flow in a rat embolism model with only one-fifth of the regular tPA dose. Huang et al. produced tPA functionalized and polyacrylic acid (PAA)-coated NPs that enabled accelerated and targeted thrombolysis and reduced infarct area in a mouse model of cerebral embolism [62]. In the work of Xie et al., so-called biomimetic magnetic microrobots (BMMs) loaded with tPA were fabricated from aligned IONP chains embedded in a non-swelling microgel shell [63]. The BMMs were capable of delivering and releasing thrombolytic drugs via magnetic guidance, which would be advantageous for minimally invasive microvascular thrombolysis. A potential treatment of in-stent thrombosis in coronary arteries was demonstrated in an in vitro study by Kempe et al. Under flow conditions, tPA-functionalized IONPs were efficiently bound to the surface of a ferromagnetic coiled wire [64].

Peptide-based targeting of tPA-functionalized IONPs was shown in other studies. In a murine model of venous and arterial thrombosis, Erdem et al. demonstrated the targeting ability of dextran-coated IONPs which were conjugated with the targeting peptide FXIIIa and functionalized with tPA [65]. In another study, IONPs were prepared from PLGA with embedded IONPs and tPA surrounded by a chitosan film onto which cRGD was grafted. The nanoparticles accumulated specifically at the edge of the thrombus and achieved a significant thrombolytic effect [66].

Besides tPA, there are other endogenous substances such as streptokinase (SK), heparin, urokinase (UK) and nattokinase (NK) that exhibit potent thrombolytic effects. An experimental thrombolytic therapy was developed by Tadayon et al. via co-functionalization of silica-coated NPs with tPA and SK [67]. The SiO_2_-NP-tPA-SK particles showed increased blood stability as well as increased storage stability in buffer. In addition, the thrombolysis efficiency could be significantly increased by magnetic targeting. A heparin delivery platform composed of IONPs, polyethylenimine (PEI) functionalized black phosphorus nanosheets, and heparin loading demonstrated accurate magnetic enhancement capacity and offers a promising multifunctional strategy to prevent deep vein thrombosis in patients at high risk of thrombosis [68]. Thrombolysis efficiency of UK can be improved by magnetic control of UK-coated IONPs [69,70]. After injection of IONPs and UK into an in vitro thrombus model, a static magnetic field created elongated NP clusters that were forced into rotation by a rotating magnetic field [71]. The generated vortex enhanced the diffusion of UK to the thrombus surface, thus accelerating its dissolution. Another study using experimental clots demonstrated the thrombolytic efficacy of IONPs functionalized by heparin-mediated cross-linking of urokinase [72]. NK-conjugated IONPs showed delayed NK release and could be targeted into the thrombus under the control of an external magnetic field or by RGD targeting [73,74].

In addition to the strategies already presented, other technologies are being developed and explored to find the most effective and rapid treatment of thrombi. Sonothrombolysis represents a technical development to increase thrombolysis efficacy. Magnetic microbubbles can be retained and set in vibration by a rotating magnetic field in the target region and then activated by ultrasound to significantly increase the lysis of blood clots [75,76]. In a mouse model of venous thrombosis, a combination of external magnetic field and high-intensity ultrasound allowed porous magnetic microbubbles loaded with tPA to be targeted to the thrombi, efficiently delivering the tPA into the clot and significantly accelerating thrombolysis [77]. In another study, multifunctional NPs were prepared that, in addition to functionalization with the EWVDV peptide that recognizes the P-selectin of thrombi, also allowed multimodal imaging by photoacoustics, magnetic resonance and ultrasound. Focused ultrasound irradiation, which induced a phase transition of NPs, enabled effective thrombolysis [78]. Focal hyperthermia may also lead to improved thrombolysis. In a rat embolism model, controlled release of tPA from thermosensitive magnetoliposomes was achieved by thermodynamic and magnetic manipulation, thereby efficiently restoring iliac blood flow [79]. Another technique to resolve thrombi was demonstrated in the study by Jeon et al., in which the combination of a traversing proton beam and IONPs caused a thrombolytic effect in an arterial thrombosis mouse model [80]. The technique could potentially be applicable in cases where treatment with tPA is not possible or ineffective.

### 3.2. Vascular Grafts and Stents

Despite the continuous improvement in biocompatibility and performance of cardiovascular grafts made from biomaterials, their successful translation into the clinic is very slow [38,81]. The reasons for this are manifold. For one, efficacy and safety must be demonstrated in vitro and in vivo in relevant animal models and confirmed in clinical trials. So far, many materials have demonstrated promising mechanical, chemical and physical properties for biomedical applications leading to the development of a wide range of nanostructured copolymers for cardiovascular grafts and stents [82,83]. Moreover, the incorporation of IONPs in biomaterials enables magnetic-based non-invasive imaging to monitor the position and performance of tissue-engineered constructs after implantation. This ought to minimize the likelihood of unexpected effects in clinical trials, shorten the regulatory pathway, and increase the commercial appeal and profitability of the therapy when translated from the laboratory to the clinic. Finally, as incomplete endothelialization leads to an increased likelihood of thrombosis formation and anastomotic intimal hyperplasia, vascular grafts and stents can be manufactured with a protecting endothelial cell (EC) layer or with an enhanced capability to be endothelialized in vivo post implantation [84].

However, it should be noted that surgical interventions to restore blood flow, whether by percutaneous transluminal angioplasty, endarterectomy, bypass grafting or even vascular prostheses or stenting, often lead to another blockage called restenosis. For this reason, more studies have now been conducted on anti-restenosis drug delivery systems based on nanomaterials, including the utilization of IONPs [85].

#### 3.2.1. IONP-Based MRT Monitoring of Grafts and Stents

Cellular coating of tissue-engineered vascular grafts (TEVGs) with IONP-labeled cells or embedding of IONPs into the scaffold itself has been shown to be an efficient method for visualizing TEVG function and localization using MRI. One of these studies evaluated ways to reduce the production of reactive oxygen species (ROS) often induced by IONPs [86]. For this purpose, the authors coated artificial ePTFE vascular grafts with poly(1,8-octamethylene citrate) (POC) as an antioxidant coating and seeded the scaffolds with ECs pre-labeled with chitosan-coated IONPs. This approach allowed in vitro MRI monitoring of the endothelium on artificial vascular prostheses without increased oxidative stress.

In the study by Luderer et al., biodegradable scaffolds made of a polymer blend of poly(l-lactide) (PLA)/poly(4-hydroxybutyrate) in which gold, silver and magnetite nanoparticles have been embedded were investigated as a replacement for permanent metallic stents [87]. In X-ray experiments, the NP-modified polymers showed improved scaffold material visibility. While scaffolds with rod-like gold nanoparticles visualized well in the near-infrared region at 820 nm, IONPs enable enhanced visualization by MRI.

In another study including in vivo experiments, ultra-small IONPs were incorporated into polyvinylidene fluoride (PVDF)-based textile fibers that were knitted into vascular scaffolds. The scaffolds were then seeded with a composite of fibroblasts, smooth muscle cells (SMCs) and fibrin, and the inner lumen was seeded with ECs in a bioreactor to prevent inflammation and thrombus formation. The functionality of the grafts, as well as their suitability for MRI were successfully verified after implantation in sheep as arteriovenous shunts between the carotid artery and the jugular vein [88]. In another publication, the group evaluated the feasibility of using multimodality imaging modalities, such as MRI and positron emission tomography–computed tomography (PET-CT), to further assess the function of their TEVGs [89]. The combined data from MRI, to monitor graft localization and function, and FDG-PET-CT, to identify and ultimately accurately quantify vascular inflammation, revealed comprehensive quality control of TFVGs in the sheep model, with no calcification, negative effects on extracellular matrix (ECM) or endothelialization.

An interesting study by Harrington et al. took the opportunity of MRI imaging to investigate the fate of cells on TEVGs [90]. Biodegradable polyglycolic acid-based scaffolds sealed with poly-ε-caprolactone (PCL) and PLA were seeded with ultra-small IONP-labeled murine macrophages and implanted into mice as inferior vena cava interposition grafts. Serial MRI studies demonstrated rapid loss of seeded cells. The findings demonstrated the need for imaging techniques to monitor and verify the functionality of tissue engineered products. In contrast to the aforementioned publication, Nelson et al. achieved non-invasive monitoring of ultra-small IONP-labeled human aortic SMCs and human aortic endothelial cells (hAECs) incorporated into TEVGs of PLA nonwoven felts. These scaffolds, coated with a copolymer of PCL and PLA were implanted into mice as aortic interposition grafts and monitored for 3 weeks by MRI [91].

#### 3.2.2. IONP-Based Improvements of Vascular Scaffolds

There are a variety of materials that can be used for TEVG production. These include hydrogels, polymers and nanomaterials, which may not only act as a support structure and provide a compatible surface for cells, but also release substances when required and promote and accelerate tissue formation.

Karbastian et al. fabricated artificial blood vessels reinforced with carbon nanotubes and IONPs from biodegradable polyurethane (PU)-thermoplastic-elastin compounds that exhibited efficient tensile strength and reasonable flexibility [92]. Another study evaluated a strategy to improve the adhesion of ECs to various membranes of bacterial cellulose (BC) by applying oscillating magnetic fields [93]. Compared with BC and magnetic BC, ECs on RGD peptide-grafted magnetic BC membranes generally showed better cell adhesion and proliferation that is further enhanced by applying a low magnetic field frequency of 0.1 Hz. Magnetic BC with embedded IONPs was also used by Arias et al. for TEVG production [94]. The embedded IONPs were protected from oxidation by an additional dextran coating. Magnetization of the resulting composite hydrogel allowed magnetically-functionalized SMCs to be retained under dynamic flow conditions and form a biologically active dense cell layer.

In contrast to the aforementioned strategy of capturing IONPs-loaded cells by magnetizable scaffolds, the work of Perea et al. demonstrated another possibility [95,96]. IONPs-loaded ECs or human SMCs were uniformly applied by a radial symmetric magnetic force to the lumen of PTFE or collagen scaffolds placed at the center of a ring-shaped electromagnet. By this method, scaffolds could be rapidly and efficiently fabricated with multiple cell layers. The same technique was used to endothelialize tubular 3D electrospun scaffolds composed of a mixture of PCL and silk fibroin, combining the mechanical advantages of PCL with the enhanced support of cell attachment provided by silk fibroin [97]. In another work, a cylindrical magnet was inserted into the lumen of decellularized porcine common carotid artery (dCCA) and immersed in a suspension of magnetically labeled murine 3T3 fibroblasts. Magnetic cell seeding resulted in effective cell colonization, which was also successfully performed with human SMCs and dermal fibroblasts [98]. Fayol et al. prepared polysaccharide-based and gelatin-coated porous scaffolds [99]. Efficient cellular coating was enabled by a magnet-based technique in which human ECs or endothelial progenitor cells (EPCs) were labeled with IONPs and then magnetically directed into the lumen, where they attached and formed a continuous endothelium. Pluricellular TEVGs could be achieved by pre-incorporation of mesenchymal stem cells (MSCs) into the pore structure and subsequent magnetic cell seeding with IONPs-labelled ECs. Another study used a Halbach cylinder device to rapidly direct IONP-loaded fibroblasts to the luminal surfaces of large tubular constructs [100]. Compared with the dynamic rotation technique, in which cell distribution was highly irregular, the distribution of cells achieved by the magnetic method was much more uniform. This study demonstrates the advantages of using radially symmetric and homogeneous magnetic fields and IONPs-loaded cells over conventional methods in colonizing tubular structures.

#### 3.2.3. IONP-Based Stent Improvements

Stent implantation is one of the most common invasive treatments for patients with coronary artery disease. Meanwhile, a variety of commercial stents and experimental stents made of different materials are available, based on polymer-free metal platforms, biodegradable/bioresorbable polymers, as well as stents that allow drug delivery by magnetic nanoparticles [101,102]. Since accelerated endothelialization may lead to a reduced incidence of restenosis or thrombosis not only in TFVGs but also in stents, rapid endothelialization is therefore of great importance to ensure long functionality and minimize complications [103]. Consequently, stents made of magnetic and biocompatible materials capable of attracting and retaining IONP-labeled ECs are advantageous [104,105,106,107].

In one study, a biocompatible polymer-free composite coating of magnetic mesoporous silica nanoparticles (MMSNs) and carbon nanotubes (CNTs) was prepared, which had a sufficient drug (rapamycin) release performance, and the advantage of rapid endothelialization compared with other commercial polymer-coated drug-eluting stents [108]. Lee et al. coated magnesium stents with biodegradable PLA polymer with embedded magnetizable iron-platinum (FePt) NPs [109]. These stents allowed efficient and uniform capture of IONPs-labeled progenitor stem cells even under flow conditions. Tefft et al. demonstrated rapid in vivo endothelialization of novel stents made of magnetizable duplex stainless steel (2205 SS) [110]. By labeling autologous blood-derived ECs with PLGA-coated IONPs and administering them intracoronary, the stents, which were additionally magnetized by attaching two 1.0-T permanent magnets to the chest wall of the pigs, were extensively endothelialized within 3 days. In another work, Adamo et al. demonstrated the feasibility of rapid and efficient in vivo endothelialization of stented carotid arteries in a rat carotid model after stent angioplasty by magnetically guided delivery of IONP-loaded ECs [111]. In a rat carotid artery stent angioplasty model, the benefits of magnetically assisted delivery of ECs to prevent vascular lumen narrowing after stent angioplasty were demonstrated by Polyak et al. [112]. Magnetic cell colonization at the distal end of the stented artery showed significant protection against stenosis after 2 months compared with the proximal part of the stent.

Targeted cell delivery was shown by Chen et al. by using anti-CD34-conjugated IONPs with high affinity for EPCs. Magnetization of iron stents resulted in a high adsorption of IONPs and magnetically labeled EPCs, demonstrating that this method can efficiently promote EPC capture and endothelialization of iron stents [113].

IONPs could also be used to functionalize stents with antirestenotic drugs or antiplatelet agents to prevent stenosis and thromboembolic complications until the metallic surface is covered with a neo-EC layer [114,115]. Furthermore, a repeatable, targeted, and patient-specific drug delivery by drug-coupled IONPs and magnetizable stents is another promising procedure for the treatment of restenosis [107,116]. Räthel et al. coupled rapamycin, which has an antiproliferative activity, to IONPs and then incorporated them into lipid-based microbubbles. In vitro flow-through experiments demonstrated successful trapping of the microbubbles by an external magnet and increased deposition on the stent struts of nickel-plated commercial stents [117]. Other studies used IONPs with paclitaxel (Ptx) to prevent SMC migration and proliferation. Johnson et al. fabricated biodegradable PLA/PLGA-based paclitaxel (Ptx) and IONP-loaded nanoparticles that potentially could allow re-dosing of depleted drug-eluting stents, thereby extending the life of the implant [118]. In another study, Chorny et al. demonstrated the feasibility of site-specific delivery of paclitaxel (Ptx) to implanted magnetizable stents in a rat carotid stenting model by uniform magnetic field-guided targeting of Ptx-loaded IONPs that resulted in significant inhibition of in-stent restenosis [119]. Finally, to treat in-stent restenosis after stent implantation, Wang et al. developed Ptx-loaded PLGA nanoparticles embedded in IONP-coated microbubbles. After magnetic targeting to the stents, focused low-intensity ultrasound induced microbubble vibration and release of PLGA-Ptx [120].

### 3.3. Atherosclerosis

Despite immense diagnostic and therapeutic advances, atherosclerosis remains a global public health problem and will continue to require the development of new strategies. These new approaches are increasingly based on molecular understanding of the disease, allowing specific nanomedicine-based molecular imaging and therapy [121,122,123,124,125,126]. Of particular interest are IONPs that can be used as transport vehicles for diagnostic or therapeutic agents through specific surface coating and functionalization, thus enabling site-specific and targeted delivery [127,128,129,130]. Meanwhile, there are numerous studies on NP-based imaging of plaques [131,132]. Among these, contrast-enhanced MRI plays a dominant role and reports demonstrate that MRI with IONPs is suitable for detailed mapping of biomarker expression in lesions compared with other contrast agents such as gadolinium [133,134].

#### 3.3.1. IONP-Based Atherosclerosis Imaging

Imaging of atherosclerotic plaques is critical for diagnosis, assessment of treatment modality and monitoring of therapy progress. Numerous studies have shown that the use of IONP and its targeted delivery to atherosclerotic plaques are able to provide accurate imaging. In the following, some studies are described in which IONP were specifically applied for the detection of plaque-associated cells or cell components, such as macrophages, foam cells, monocytes, ECs, platelets, or for the detection of other potential biomarkers of atherosclerotic plaques.

A large number of studies have reported the imaging of atherosclerotic plaques by labelling of macrophages with IONPs. In particular, the use of ultra-small IONPs or very-small IONPs particles for the detection of macrophages and pathological inflammation in atherosclerotic tissue is well established [135,136,137,138,139,140,141]. Especially, ferumoxytol (Feraheme^®^) and ferumoxtran-10 (Sinerem^®^/Combidex^®^), which have an excellent safety profile and are selectively taken up by macrophages, has been widely reported [142,143,144,145,146,147,148,149,150,151]. In a recent study, ultra-small IONPs was tested for potential high-risk atheroma imaging in which nanoparticles deposited in plaque macrophages, SMCs and ECs and associated with areas of plaque neovascularization and impaired surface endothelial permeability [152]. However, the slightly larger IONPs are also suitable for the imaging of atherosclerosis [153] and a clinical study demonstrated the potential suitability of fercarbotran for detecting infiltration of macrophages in human atherosclerotic carotid plaques [154].

To improve the labeling efficiency of macrophage-rich plaques, IONPs can be functionalized with substances for increasing the IONP accumulation or targeting efficiency. Nakamura used heparin-modified calcium phosphate nanoparticles loaded with ferucarbotran (Resovist^®^) and showed higher delivery of iron oxide in form of composite nanoparticles to macrophage-rich carotid artery lesions in mice compared to free ferucarbotran [155]. Other studies used IONP with dextran, a ligand of macrophage scavenger receptor type A (SR-A) for macrophage-targeted MRI. For example, IONPs coated with dextran sulfate showed significant signal loss at the injured carotid artery after intravenous injection into an atherosclerotic mouse injury model [156]. Another study used oleyl-dextran-coated magnetic nanoclusters for the detection of macrophage-rich atherosclerotic plaques in a rat arterial balloon injury model [157]. After intravenous injection of PEG-coated and DNA oligonucleotide-coupled IONPs in an apolipoprotein E knockout (ApoE^−/−^) mouse model, DNA-IONPs were shown to accumulate effectively in macrophages of atherosclerotic plaques, most likely by binding to SR-A and lipid rafts, thus providing an effective strategy to enhance systemic delivery of NP to atherosclerotic plaques [158]. In vivo recognition of macrophages in atherosclerotic carotid arteries could also be achieved with IONPs provided with a ferritin protein shell [159]. Segers et al. used ultra-small IONPs with the SR-AI peptide ligand PP1 (LSLERFLRCWSDAPAK) to achieve higher iron uptake in macrophages for improved visualization of atherosclerotic plaques in ApoE^−/−^ mice [160]. In another study, Kitagawa et al. demonstrated effective MRI detection of vascular inflammation and angiogenesis in carotid disease and abdominal aortic aneurysm by RGD-conjugated iron oxide nanoparticles and their specific targeting of macrophages and angiogenic ECs [161]. There are also reports of antibody-based targeting. Tarin et al. used gold-coated IONPs coupled with an anti-CD163 antibody to enable targeted detection of the CD163 receptor, which expression is increased in macrophages at inflammatory sites [162]. Finally, Ji et al. developed anti-CD68 receptor-targeted Fe-doped hollow silica nanoparticles as a multimodal ultrasound/MRI contrast agent to identify macrophages in atherosclerotic plaques in ApoE^−/−^ mice [163].

Foam cells, or lipid-laden macrophages, are cholesterol-containing cells which play a critical role in the occurrence and development of atherosclerotic plaques and are induced by several factors, such as imbalance of cholesterol influx, esterification and efflux [164]. Wu et al. fabricated magnetic mesoporous silica NPs with near-infrared fluorescence (NIRF) dye (IR820) and PP1 peptide, a targeting peptide that binds to the surface receptor (SR-AI) on foam cells, to detect macrophage accumulation in atherosclerotic plaques of ApoE^−/−^ mice by dual MR/NIRF imaging [165]. In another study, annexin V, which binds to apoptotic cells such as foam cells of atherosclerotic plaques by interaction with phosphatidylserine, was bound to IONPs and administered parenterally to rabbit models of human atherosclerosis [166]. Annexin V-IONPs distributed rapidly and deeply into early apoptotic plaque foam cells and enabled a biologically targeted MRI detection and evaluation of cardiovascular lesions and the differentiation between occlusive and mural plaques. Annexin V was also utilized in another study to produce an ultra-small IONP-based multimodal nanoparticle system for single photon emission computed tomography (SPECT)/ MRI to detect apoptotic macrophages in vulnerable plaques [167].

An early marker of atherosclerotic plaque formation is the migration of monocytes from the circulation into the beginning lipid accumulation in the arterial wall. IONPs coupled with a monocyte chemoattractant protein-1 (MCP-1) peptide motif accumulated in the aorta of atherosclerosis model mice that exhibited monocyte accumulation, and thus could serve as a diagnostic tool for atherosclerosis [168].

While the healthy endothelium has a protective function, activation and dysfunction of endothelial cells affects, among other things, leukocyte adhesion and recruitment, platelet activation and thrombus formation, ultimately promoting the formation of atherosclerotic plaques [126]. Endothelium-directed NPs can therefore be used in combination with imaging methods to visualize structures and activities of the atherosclerotic endothelial wall. Kelly et al. developed targeted IONPs for MRI imaging that were coupled with the phage display-derived peptide sequence VHSPNKK and could bind to endothelial vascular adhesion molecule-1 (VCAM-1), a critical component of the leukocyte-endothelial adhesion cascade, and thus be taken up by VCAM-1-expressing cells [169]. In vivo, IONPs identified VCAM-1-expressing ECs in a murine tumor necrosis factor-alpha-induced inflammation model and colocalized with VCAM-1-expressing cells in atherosclerotic lesions present in cholesterol-fed ApoE^−/−^ mice. In similar studies, magnetic NPs modified with VHPKQHR peptides were used for targeting VCAM-1-expressing cells [170], which could in principle be loaded with therapeutic agents and thus be suitable for drug delivery [171]. In the study by Michalska et al., the use of ultra-small IONPs functionalized with VCAM-1-binding peptide (P03011) resulted in a distinct visualization of the aortic root of ApoE^−/−^ mice [172]. Histological analysis confirmed iron accumulation in the intima, in colocalization with VCAM-1-expressing macrophages and ECs.

For early detection of atherosclerotic plaques and activated platelets, which play a central role in thrombosis, atherosclerosis and inflammation, Prévot et al. coupled the platelet-specific scFv-Fc TEG4-2C antibody with a IONP-containing magnetic oil-in-water nano-emulsion and achieved significant labeling of atheroma plaques both in vitro and ex vivo in animal models using magnetic particle imaging (MPI) and MRI [173]. Specific targeting of platelets was also achieved by using a single chain antibody (scFv) [174,175]. Coupling of scFv to IONPs or cells provided the possibility of molecular imaging and cell homing in cardiovascular and inflammatory diseases. In another study, rhodamine-labeled PEGylated dextran/IONPs were used in conjunction with an anti-human P-selectin antibody to target P-selectin, which is expressed at high levels on activated platelets and ECs, with high affinity to identify the early stages of atherosclerosis [176]. A successful targeting of activated platelets within atherosclerotic lesions was also achieved by ultra-small IONPs functionalized with a recombinant human IgG4 antibody (rIgG4 TEG4) [177].

There are abundant biomarkers suitable for NP-based homing to atherosclerotic plaques. One study used hybrid metal oxide-peptide amphiphile micelles (HMO-Ms), which are designed to enable MRI imaging of thrombosis at atherosclerotic plaques through the fibrin-targeting sequence CREKA [178]. In vivo studies in a murine ApoE^−/−^ plaque model using poly(maleicanhydride-alt-1-octadecene) (PMAO)-coated IONPs showed that the IONPs accumulated in similar vascular regions as an elastin-targeting gadolinium-based contrast agent which accumulated in plaques [179]. Kim et al. compared the potency of the two targeting ligands, cRGD peptide and collagen IV, which binds to αvβ3-integrin overexpressed in neovasculature and collagen type IV present in plaque, respectively [180]. Of the two IONP-conjugated targeting ligands, cRGD-based targeting was more efficient than collagen IV targeting peptide in the early stage of atherosclerosis in the Apo E-/- mouse model. Targeting of atherosclerosis was also successfully achieved with spherical nanocomplexes of zinc-doped ferrite nanoparticles, bovine lactoferrin, PEG, and Hsp-70 antibodies [181]. Histological studies after injection into a Psammomys obesus model of type 2 diabetes, obesity and atherosclerosis confirmed site-specific accumulation at the atherosclerotic aortic arch and descending thoracic aorta of animals with severely damaged intima full of ruptured microatheromas. Multimodal molecular imaging of atherosclerotic plaques from ApoE^−/−^ mice was similarly enabled by profilin-1-targeting IONPs, through conjugation of a polyclonal profilin-1 antibody and an NHS-Cy5.5 fluorescent dye [182]. Wie et al. used IONPs functionalized with the fusion protein ‘enhanced green fluorescent protein with the first epidermal growth factor domain’ (EGFP-EGF1) to detect tissue factor (TF)-positive atherosclerotic plaques in ApoE^−/−^ mice [183].

Another approach to detect atherosclerotic plaques is the use of IONPs with high-density lipoproteins (HDL) [124]. After injection of these particles into ApoE^−/−^ mice, the iron oxide nuclei were found in large amounts within the atherosclerotic plaques of the aorta and thus penetrate into the plaques, similar to native HDL. In a similar study, multimodality imaging confirmed that HDL-labeled IONPs and quantum dots accumulated in atherosclerotic lesions in mice after intravenous and especially after intraperitoneal injection [184]. Another study used polyethylene glycol (PEG)-coated ultra-small IONPs conjugated with polyclonal anti-mouse oxidized low-density lipoproteins (OxLDL) antibody for direct detection of OxLDL and imaging of atherosclerotic lesions in ApoE^−/−^ mice [185]. Li et al. used β-cyclodextrin-conjugated IONPs for potential molecular imaging of crystallized cholesterol in atherosclerotic plaques [186].

A further study demonstrated that tenascin-C, a multifunctional extracellular glycoprotein that is highly expressed in advanced atherosclerotic plaques and is associated with inflammatory changes and plaque rupture, can serve as a marker for atherosclerotic plaques [187]. After injection of anti-tenascin C-ultra-small IONPs, MR images correlated well with histopathological analysis and the progression of atherosclerotic plaques. IONPs can not only be used for plaque detection, but are also suitable for assessing the condition of plaques. Embedding perfluorooctyl bromide and IONPs in PLA and linking vascular endothelial growth factor receptor-2 (VEGFR-2) antibody on the surface of the particles enabled bimodal MRI and ultrasound visualization of intraplaque neoangiogenesis, which is a biomarker for impending plaque rupture [188]. Atherosclerotic plaques could also be detected by using hyaluronan-conjugated iron oxide nanoworms (hyaluronan-NWs). Compared with spherical hyaluronan-coated nanoparticles, hyaluronan-NWs bind more strongly to CD44, a cell surface protein overexpressed in plaque tissues, and enabled the non-invasive plaque detection by MRI in an ApoE^−/−^ mouse model [189]. GEBP11-peptide-targeted IONPs showed good visualization of angiogenesis in atherosclerotic plaques after intravenous injection in a rabbit model of atherosclerosis, which may be useful for molecular imaging of progressive plaque angiogenesis leading to plaque hemorrhage and vulnerability [190]. In the work of Tong et al., multimodal NPs were developed by conjugating IONPs with 5-hydroxytryptamine and cyanine 7 N-hydroxysuccinimide ester to detect active myeloperoxidase (MPO), a potential inflammatory marker of vulnerable atherosclerotic plaques [191]. MPI, fluorescence imaging (FLI), and computed tomographic angiography (CTA) in an ApoE^−/−^ mouse model confirmed high specificity and sensitivity of the particles and allowed quantitative assessment of the degree of inflammation. Finally, to identify vulnerable plaques and rupture plaques, dimercaptosuccinic acid (DMSA) was bound to ultra-small IONPs and showed high specificity and sensitivity for early detection of vulnerable plaques and ruptured plaques after injection into an atherosclerotic rabbit model [192].

#### 3.3.2. IONP-Based Therapy of Atherosclerosis

IONP not only enable the imaging of artherosclerotic plaques, but can also serve as vehicles for therapeutic agents. The efficacy of drug delivery is mostly achieved by passive aggregation provided by the enhanced permeability and retention effect. Active drug delivery can also be achieved using targeting molecules, or specific stimuli such as magnetic fields or ultrasound, to effectively target atherosclerosis at the molecular level [193]. The study by Banik et al. presented a theranostic nanoparticle platform with mitochondria- and macrophage-targeted surface functionalities that lowered lipid levels in the body without causing a significant immunogenic effect [194]. The presence of a mannose-bearing ligand also allowed targeting of macrophages normally present in atherosclerotic plaques. Zhang et al. used theranostic composite IONPs containing non-inflammatory cyclodextrin, a profilin-1 antibody, and the anti-inflammatory drug rapamycin to target vascular SMCs in atherosclerotic plaques and inhibit the progression of atherosclerosis in ApoE^−/−^ mice [195]. Another theranostic approach to plaque treatment, based on the expression of alpha(v)beta3-integrin by the vasa vasorum, was described in the study by Winter et al. [196]. Administration of fumagillin, an antiangiogenic agent, with ανβ3-integrin-targeted paramagnetic nanoparticles allowed the quantitation of angiogenesis and inhibition of the proliferation of the vasa vasorum in hyperlipidemic rabbits.

Reactive oxygen species (ROS) play an important role in inflammatory reactions such as those associated with atherosclerosis. In an in vitro study with theranostic Fe_3_O_4_/CeO_2_ core-shell nanoparticles, it was shown that they can effectively capture ROS and are well detectable by MRI, making them potentially suitable for the treatment and diagnosis of ROS-related inflammatory diseases [197].

Another theranostic strategy for vulnerable plaques using an adaptable nanoparticle platform was demonstrated in the work of Bonnet [198]. They developed a PEG-coated nanoemulsion (NE) functionalized with human scFv-Fc antibody and loaded with IONPs and an active pharmaceutical ingredient (alpha-tocopherol). Targeted antibody recognition of galectin 3, an atherosclerosis biomarker and reduction of oxidation by alpha-tocopherol could potentially reduce the risk of plaque rupture. Another group used PEG-coated ultra-small IONPs coupled with antibodies against connective tissue growth factor (CTGF) as well as free anti-CTGF to recognize and neutralize CTGF within atherosclerotic lesions of mice [199]. While anti-CTGF-treated animals exhibited reduced macrophage deposition, CTGF expression, and plaque volume, future experiments are needed to verify the effect of the NPs.

The combination of IONPs with the phase transition material perfluorohexane and with dextran sulfate (DS) targeting SR-A enabled specific targeting of activated macrophages in an atherosclerotic plaque model of ApoE^−/−^ mice [200]. Low intensity focused ultrasound irradiation (LIFU) could trigger the induction of apoptosis in macrophages with endocytosed NPs, allowing theranostic treatment of atherosclerosis. Oumzil et al. demonstrated that solid lipid nanoparticles loaded with IONPs and the therapeutic agent prostacyclin (PGI2) can inhibit platelet aggregation and also exhibit very good relaxation properties for MRI imaging [201]. Finally, Gao et al. presented a strategy to distinguish and treat rupture- or erosion-prone plaques [202]. PLGA-NPs containing IONPs and perfluoropentane coated with PP1 and cRGD peptides, enabled plaque characterization by ultrasound and MRI. In addition, the NPs might also promote therapeutic effects through the ultrasound-induced phase change from nanodroplets to gaseous microbubbles. Theoretically, by ultrasound, the NPs could induce apoptosis in macrophages via binding of PP1 to SR-A, thereby reducing chronic infiltration of inflammatory cells into rupture-prone plaques. The NPs could also target erosion-prone plaques, through binding of cRGD to glycoprotein (GP) IIb/IIIa on activated platelets, and promote platelet disaggregation by ultrasound.

#### 3.3.3. Magnetic Drug Targeting to Atherosclerotic Plaques

Targeted enhancement of IONPs can be achieved by the presence of local magnetic fields, promising enhanced and improved diagnosis of inflammatory plaques by MRI. By applying an external magnetic field, Shi et al. achieved a slowdown of IONPs in blood flow, alteration of their trajectory, and ultimately efficient uptake into inflammatory cells, resulting in a clear visualization of plaques via MRI [203]. Similar to the magnetically enhanced plaque detection, drug-loaded IONPs can be directed and retained at the desired location by magnetic fields for theranostic approaches [204]. Magnetic drug targeting (MDT) thus realizes higher concentrations of bioactive molecules at the target site and mitigates potential systemic side effects. In proof-of-concept studies from our group, magnetic accumulation of circulating IONPs in the non-uniform shear stress region of a bifurcating flow model was investigated by Matuszak et al., showing the applicability of magnetic targeting of arterial-like geometries [205,206]. In a further study of our group, dexamethasone phosphate (Dexa)-functionalized IONPs were directed into the abdominal aorta of an atherosclerosis rabbit model by MDT [207]. Although the treatment did not produce the expected anti-inflammatory results, the study demonstrated good targeting efficacy that has the potential to provide an efficient treatment of atherosclerotic plaques with other IONP-coupled drugs, such as statins.

#### 3.3.4. Cell-Based Plaque Regeneration

Apart from pure IONP-based treatment of atherosclerotic plaques, cells, especially EPCs, may also be used for therapeutic angiogenesis and vascular repair and, after loading with IONPs, to monitor the cell migration into vessels. For instance, after transplantation of ultra-small IONP-poly-l-lysine-labeled EPCs into an atherosclerotic rabbit model, results suggest that ultra-small IONP-labeled EPCs may play a role in repairing endothelial damage and preventing atherosclerosis [208].

### 3.4. IONPs as Modulator and Enhancer of Cardiovascular Regeneration

Currently, there are a variety of pharmaceutical drugs for the treatment of cardiovascular diseases, including calcium channel blockers, antioxidants, oxygen free radical scavengers and anti-apoptotic agents, but they are often associated with side effects. The use of IONPs as vehicles for targeted drug delivery could significantly reduce systemic effects. Moreover, NPs can either be coupled with drugs or other active substances or enable gene transfer for overexpression or silencing of relevant genes by binding DNA or RNA. Xiong et al. have demonstrated cardio protective activity of DMSA-IONPs after intravenous injection [209]. The IONPs were effective in protecting against ischemic damage and also exhibited no significant toxicity toward cardiomyocytes. However, the exact underlying mechanisms for the cardioprotective effect were uncertain.

Functionalized IONPs can also be used to reduce leukocyte migration and thereby attenuate inflammatory responses. In one study, siRNA against chemokine (C–C motif) receptor 2 (CCR2), a chemokine receptor critical for leukocyte migration, was encapsulated in nanoparticles to treat inflammatory cell infiltration of the heart and subsequent deterioration of cardiac function in myocarditis [210]. CCR2 silencing in mice with acute myocarditis reduced the migration of granulocyte-macrophage progenitor cells from the bone marrow into the blood, suggesting that this strategy may indicate a pathway for successful treatment of myocarditis. It should be mentioned that nanoparticles can be used not only to suppress inflammation, but also for its detection. After intravenous application of ultra-small IONPs, they will be absorbed by macrophages. As macrophages and other immune cells accumulate at the site of inflammation, they can be detected by MRI and, for example, enable the early detection of rejection reactions, e.g., of transplanted hearts [211]. After intravenous injection of magnetic nanobeads functionalized with adenoviral vector-encoded human vascular endothelial growth factor (hVEGF) gene, Zhang et al. achieved a strong therapeutic gene expression and significantly improved function in ischemically injured hearts of rats with acute myocardial infarction by magnetic targeting [212].

Alternative approaches for magnetically-based targeted drug delivery are currently under intense investigation, as this technique is expected to enable the local enrichment of IONPs and improve the therapeutic outcome by specifically enhancing tissue production. IONPs externally navigated by applied magnetic fields could help to regain tissue function, e.g., in the treatment of cardiac arrhythmias [213]. Improved cardiac function in systolic heart failure rat models was achieved by Kiaie et al. by loading chitosan-coated IONPs with the cardiac myosin activator omecamtiv mecarbil and targeted delivery using an external magnet to the rats’ hearts [214]. Sivaraman et al. focused on localized elastic matrix stabilization and regenerative repair [215]. An underlying cause of the growth of abdominal aortic aneurysms is chronic overexpression of matrix metalloproteases (MMPs), which destroy the elastic matrix in the aortic wall while further decreasing the poor autoregeneration of these matrix structures. One way to increase elastic matrix deposition and to inhibit MMPs is by sustained administration of doxycycline from PLGA NPs [215]. In a further publication, the same group demonstrated increased elastic matrix deposition and significant inhibition of MMP synthesis and activity by controlled targeting of PLGA NPs containing doxycycline and IONPs by an applied external magnetic field [216].

As an alternative cell-free therapy, angiogenesis and cardiac function in infarcted heart tissue could be enhanced by the accumulation of extracellular vesicles, which are necessary to maintain tissue homeostasis. To this end, Liu et al. used SiO_2_-coated and PEG-decorated IONPs functionalized with two antibodies, one against CD63 antigens on the surface of extracellular vesicles and one against myosin light chain surface markers on injured cardiomyocytes, and performed magnetically directed accumulation of exosomes on injured cardiac tissue in rabbit and rat models of myocardial infarction [217]. Another study demonstrated the production of IONP-loaded exosome-mimetic extracellular nanovesicles from IONP-loaded MSCs [218]. After injection into the infarcted heart, magnetic guidance significantly increased their retention, induced an early shift from the inflammatory phase to the reparative phase, decreased apoptosis and fibrosis, and enhanced angiogenesis and recovery of cardiac function. In a study by Santoso et al., cardiomyocyte-derived exosomes improved cardiac function and myocyte viability after myocardial infarction by regulating autophagy in hypoxic cardiomyocytes, which may in the future enable cell-free, patient-specific therapy for ischemic cardiomyopathy [219].

### 3.5. Stem Cell Therapy

**Figure 3 nanomaterials-11-02337-f003:**
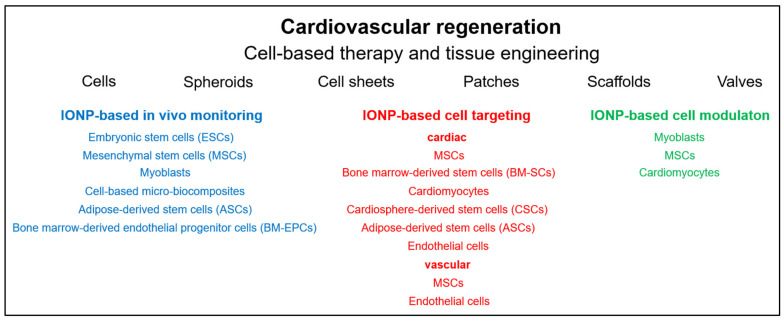
IONP-assisted cell-based therapy and tissue engineering. Shown are examples of various IONP-loaded stem cells used for imaging, cell targeting and cell modulation in cardiovascular regeneration.

Stem cell therapy has been considered the greatest hope for the treatment of cardiovascular diseases. In principle, their use could promote regeneration of all injured and diseased tissues. In the treatment of cardiovascular and cerebrovascular diseases, the promotion of angiogenesis in ischemic tissues and organs represents a particular challenge [220]. Functional restoration of blood supply depends on the restoration of functional collateral networks. To this end, most tissues have molecular mechanisms to compensate for low oxygen levels through mechanisms of vasodilation, angiogenesis, arteriogenesis, vascular remodeling and hematopoiesis. However, the inherent modulators of vascular remodeling are often unsatisfactory and must be supported by proangiogenic and arteriogenic factors, such as FGF-2 and PDGF-B. In addition, regeneration of injured blood vessels or repair of ischemic tissue can be achieved by localized cell therapy with stem cells or EPCs [221]. However, cell therapy is only effective if the cells reach the place where they are expected to fulfill their purpose and remain viable there. Therefore, the route of administration is very important as the homing of intravenously administered cells is not as effective as when the cells are delivered directly to the injured or diseased tissue or steered by magnetic attraction [222]. Nevertheless, in all cases monitoring of the treatment is extremely important. Below, IONP-assisted cell-based approaches to cardiovascular therapy and tissue engineering are discussed. Examples of various IONP-loaded stem cells used for imaging, cell targeting and cell modulation in cardiovascular regeneration are listed in Figure 3.

#### 3.5.1. IONP-Based In Vivo Monitoring

Concomitant noninvasive in vivo MRI monitoring of therapy and visual differentiation of any implanted cells or grafts from a host tissue can be achieved by labeling with paramagnetic agents, such as IONPs due to their inherent imaging properties [223,224]. However, it must be noted that the MRI signal always originates from the IONPs, regardless of the surrounding area. Consequently, extracellular particles, especially after long-term tracking of transplanted stem cells, are also detected [225]. Apart from this, it is not possible to distinguish whether the cells are still vital, have already died or that the MRI signal originates from phagocytosing cells due to clearance of dead cells and their IONP-loading [226]. Although other work confirmed the possibility of tracking IONP-labeled cells during or shortly after direct intramyocardial stem cell transplantation, due to the reuptake of IONPs by tissue macrophages, MRI cannot provide reliable information about long-term cell viability and fate of transplanted cells [227,228,229]. In another study in which IONP-labeled EPCs were injected into rat myocardium, it was shown that the long-lasting signal from the iron-positive cells was mainly due to macrophages that had taken up IONPs bound to dead cells [230]. However, there are also reports that long-term tracking of IONP-labeled primary myoblasts incorporated into fibrin glue and injected into the atrioventricular groove of rat hearts is possible for up to 1 year with µCT and MRI [231]. Analyses confirmed that the IONPs were confined to viable cells in the implant and that there was no evidence of phagocytosis of the labeled cells by macrophages or release of nanoparticles from the grafted cells. In their study, Naumova et al. evaluated the efficacy of MRI imaging of cells by cellular uptake of exogenous IONPs or by overexpression of ferritin, an endogenous iron storage protein [226]. Mouse skeletal muscle cells were labeled either by co-culturing with iron oxide particles or by overexpressing ferritin and transplanted into infarcted mouse hearts. Both approaches were found to have advantages and disadvantages. For example, ferritin overexpression showed lower signal intensity and was restricted to living cells, whereas NP labeling resulted in a comparably strong signal in all injected cells, whether dead or alive.

Despite the previous reports, the study by Chung et al. demonstrated a strategy in which long-term in vivo assessment of transplanted cells by MRI is possible [232]. For this purpose, embryonic stem cells (ESCs) were first equipped with a reporter gene to express antigens (hemagglutinin A and myc) and luciferase on the ESC surface. After transplantation, the viability of the transplanted ESCs was demonstrated in vivo by using IONP-conjugated antigen-specific monoclonal antibodies. Another study demonstrated a variable viability of transplanted stem cells. To accurately assess viability, cell localization and regenerative potential of transplanted cells, Hung et al. injected IONP- and luciferase-labeled mouse ESCs into three different zones of myocardial infarction in a mouse model [233]. Multimodality imaging demonstrated that despite decreased survival of mESCs, precise delivery into the peri-infarct region resulted in significant functional recovery of the damaged anterolateral myocardium.

Up to date several stem cells of different origins has been utilized for cardiovascular tissue regeneration. A relatively new therapeutic approach for the treatment of cardiac and other diseases is the usage of human ESCs (hESCs). For in vivo visualization of transplanted hESCs, the cells can be labeled with IONPs and tracked with MRI. In an early study, hESCs cells were loaded with dextran-coated ferumoxides (Feridex IV^®^/Endorem^®^) and injected into explanted mouse hearts as well as in vivo into the anterior left ventricular wall of rats [234]. The experiments demonstrated the feasibility of safe MRI-based in vivo tracking of transplanted hESCs cells. Skelton et al. used ferumoxytol-labeled hESC-derived cardiac progenitor cells to evaluate the distribution of cells after transplantation into the left ventricular free wall of uninjured pig hearts [235]. The localization and distribution of labeled cells could be effectively imaged even after 40 days, demonstrating the suitability of ferumoxytol as a long-term, differentiation-neutral cell labeling agent.

Safe and effective loading of stem cells with IONPs to track cardiac regenerative capacity of bone marrow-derived human mononuclear cells and C2C12 skeletal myoblasts using ferumoxides as a label has also been demonstrated after injection of the cells into rat myocardium [236]. Another study showed that DMSA-coated IONPs allowed imaging of skeletal muscle tissue-derived human myoblast cells and that the IONPs did not adversely affect basic cellular functions [237].

In the study by Salamon et al., a bimodal detection of single MSCs after uptake of carboxyfluorescein succinimidyl ester-linked IOMP was performed by histological fluorescence methods and MRI [238]. In another study, it was demonstrated that differentiation of bone marrow mesenchymal stem cells (BM-MSCs) into cardiac and neuronal lineages was unaffected by IONP-labelling [239]. Other studies investigated multimodality imaging of BM-MSCs) [240,241]. MRI and bioluminescence imaging were thus used to track IONP-, Firefly luciferase reporter gene- and fluorescently labeled cells transplanted into rat hearts in vivo, whereas fluorescence imaging accurately tracked transplanted cells only in vitro [240,241]. The transplantation of NP-labeled MSCs and BM-MSCs in rats, mice and pigs allowed simultaneous cell tracking and evaluation of cardiac function in animal models using clinical MRI devices [242,243,244]. Another study showed that allogeneic BM-MSCs, transfected with a minicircle vector encoding mutant HIF1-α and labeled with IONPs for MRI tracking, significantly reduced infarct volume and improved left ventricular function after injection into the peri-infarct of sheep undergoing coronary occlusion [245].

In the therapy of aneurysms, embolization with coils is widely used. However, the endothelial layer of the aneurysm neck often loses its integrity after embolization with the consequence that the aneurysms may reappear. MRI-sensitive and IONP-loaded bone marrow-derived EPCs (BM-EPCs) could be used to regenerate the injured endothelium by differentiating into mature ECs while monitoring the regeneration process. In a rat embolization model of abdominal aortic aneurysm, IONP-labeled BM-EPCs were shown to settle mainly in the aneurysm neck and accelerate the formation of fibrous tissue, indicating that BM-EPCs can play a crucial role in the repair and remodeling of the aneurysm neck orifice [246].

The success of vascular regeneration was demonstrated by IONP-labelled adipose-derived stem cells (ASCs) after intravenous injection into the tail vein of a mouse carotid artery injury model [247]. MRI confirmed homing of the ECs into the injured carotid tissue over the following 30 days. Transplantation of allogeneic adipose-derived regenerative cells (ARCs) is also a promising treatment for ischemic diseases. Using fluorescent ARCs (GFP-ARCs) loaded with acetylated 3-aminopropyltrimethoxysilane (APTS)-coated IONPs, Zheng et al. investigated the efficacy of therapeutic angiogenesis in an ApoE^−/−^ mouse model with hind limb ischemia [248]. Implantation of the labeled ARCs into ischemic muscles was demonstrated in vitro by immunohistochemistry and in vivo by MRI and confirmed the enhancement of neovascularization. Another study demonstrated that hypoxia is a potent stimulus for the angiogenic activity of ASCs [249]. After implantation of ex vivo hypoxia preconditioned IONP-labeled ASCs in which VEGF and HIF-1α expression was increased into the infarcted myocardium of rats, capillary density and left ventricular function were improved.

Hence, several studies have demonstrated the feasibility of tracking stem cells with incorporated IONPs. However, biological safety must also be ensured when using NPs. In this context, the work of Elkhenany et al. showed large differences in the effects of different IONPs, either uncoated or coated with starch, after labeling ASCs [250]. In particular, labeling ASCs with starch-Fe_2_O_3_ NPs improved cell migration and angiogenic potential and provided them with higher resistance against apoptosis. Hill et al. demonstrated that labeling of MSCs did not affect proliferation or differentiation ability and also provided long-term detection after injection into normal and freshly infarcted myocardium in pigs. The IONP-loaded cells even provided sufficient MRI contrast for in vivo detection in a beating heart [251].

In addition to the numerous and promising research results on monitoring stem cell-based treatment of cardiovascular diseases using IONP-loaded cells, first clinical studies have now been launched. In one trial, administration of ultra-small IONP-labeled autologous BM-MSCs after intramyocardial injection in patients with chronic ischemic heart disease was shown to be safe and the cells were detectable at the injection sites by MRI for up to two weeks after transplantation [252].

#### 3.5.2. IONP-Based Cell Targeting

The use of nanoparticles in stem cell therapy is not limited to monitoring transplanted cells, but can also be used to target cells to specific sites by specific functionalization or/and magnetic forces [223]. In this context, magnetic targeting can significantly enhance cell retention. However, the applied magnetic field strength must not be too high, as this would lead to microembolization and undermining the benefits of cell transplantation [253].

In particular, magnetic targeting has been shown to significantly increase cell homing. In a heart failure rat model, IONP-loaded MSCs could significantly improve cardiac function and myocardial hypertrophy and reduce fibrosis in the presence of a magnetic field [254]. Magnetic targeting also offers advantages in retrograde coronary venous delivery of MSCs for the treatment of cellular cardiomyoplasty due to increased cell retention [255]. The study by Ottersbach et al. demonstrated significantly increased short- and long-term engraftment rates after direct intramyocardial injection of NP-labeled cardiomyocytes and application of a magnet, ultimately leading to greatly improved left ventricular function [256].

Cheng et al. enabled targeted accumulation of cells to injured tissue using antibodies and magnetic fields [257]. IONPs were coupled with two different antibodies, one targeting CD45 on exogenous BM-MSCs and the other targeting endogenous CD34-positive cells on injured cardiomyocytes to treat acute myocardial infarction. Additionally, the targeted enrichment of therapeutic cells can be further enhanced by external magnets. The same group increased cell retention and engraftment of cardiosphere-derived stem cells (CSCs) by ferumoxytol labeling with magnetic targeting after intracoronary infusion into a rat ischemia/reperfusion model, thereby improving the therapeutic benefit [258]. Transplantation of IONP-labeled EPCs via the tail vein and guidance by an external magnet over the infarct area demonstrated increased cell retention, microvessel density and proangiogenic factor expression, significantly improved cardiac function, decreased infarct size and reduced myocardial apoptosis in rats with myocardial infarction [259]. Wang et al. showed that after intramyocardial injection of IONP-labeled ASCs and the presence of a subcutaneously implanted magnet, cardiac retention of the cells increased and improved the recovery of cardiac function in rats with myocardial infarction [260].

Dangerous vessel occlusions can be restored in the clinic with interventional balloon angioplasty. However, the injury to the vessel walls that occurs during this procedure carries the risk of restenosis and neointimal hyperplasia. In one study, magnetic cell targeting of IONP-loaded MSCs in a rabbit model was shown to result in a six-fold increase in cell retention and ultimately a reduction in restenosis [261]. The study by Vosen et al. followed a different approach to restore vascular function based on radial symmetric re-endothelialization [262]. Lentiviral vectors and IONP were used to overexpress vasoprotective endothelial nitric oxide synthase (eNOS) in ECs. The IONP-loaded cells could then be positioned on the vessel wall by magnetic fields under flow conditions. Thus, it could be shown that a combined application of IONP, cell therapy and magnetic fields can lead to a re-endothelialization and functional improvement of vessels. Precise movement of IONP-loaded cells was recently demonstrated by the use of Halbach magnets by Blümler et al. [263]. Kyrtatos et al. used a Halbach array-based magnet design to deliver ferumoxides-loaded EPCs to the site of arterial injury to reduce neointima formation by re-endothelialization [264].

#### 3.5.3. MNP-Based Cell Modulation

The modulating capacity of IONPs towards cells was investigated by Han et al. [265]. The therapeutic efficacy of MSCs in myocardial infarction is mainly dependent on the differentiation into an electrophysiological phenotype, which is determined by the active gap junctional crosstalk of MSCs with cardiac cells in co-culture. In another study, gap junctional protein Cx43 was shown to be increased in IONP-loaded myoblasts and to enhance communication with MSCs, resulting in significantly higher levels of cardiac electrophysiological biomarkers and a paracrine profile favorable for regeneration [265]. Accordingly, injection of primed MSC in rat myocardial infarction models resulted in significantly improved survival and cardiac function. Chen et al. developed multifunctional silica-IONPs that are intrinsically suited for noninvasive imaging, magnetic guidance, and as vehicles for the sustained release of insulin-like growth factor (IGF), a pro-survival agent [266]. In a rabbit ligation/reperfusion model, labeling of human MSCs increased the efficacy of cell therapy and enhanced cell survival through sustained release of pro-survival agents. The study of basic electrophysiological properties was the major focus of the work of Takanari et al. [267]. Co-cultures of cardiomyocytes and IONP-loaded skeletal myoblasts (SkMB) on multi-electrode arrays were used as an in vitro model of myoblast transplantation. Subsequently, the electrophysiological, and arrhythmogenic evaluation of the experiments indicated that transplantation of co-cultures with magnetically patterned SkMB could reduce arrhythmogenecity compared with conventional transplantation by simple cell injection.

### 3.6. Cardiac Tissue Engineering and Regeneration

Another approach to restore functional cardiac tissue is the use of biomaterials, which can be manufactured from a broad variety of components and exhibit a wide diversity of structures. To achieve long-term cell retention and thus enhance the efficacy of cell-based therapies, Blocki et al. developed injectable microcapsules of agarose, supplemented with the ECM proteins collagen and fibrin and the glycosaminoglycan-like dextran sulfate, with embedded IONP-labeled MSCs, and administered them into the infarcted heart wall [268]. In contrast to free MSCs, MSCs derived from the solubilized microcapsules were detectable in the myocardium by MRI for several weeks. In another study, ASCs were encapsulated in IONP (ferumoxides)-labeled semipermeable alginate microspheres and showed improved cell retention in the myocardium of a porcine model of myocardial infarction [269].

One promising manufacturing method is electrospinning. In one study, to improve mechanical properties and surface area, casein-coated IONPs were incorporated into electrospun silk fibroin nanofibers [270]. After seeding mouse embryonic cardiac cells (ECCs), the scaffold was confirmed to be cytocompatible, revealed no negative effects on proliferation ability, and demonstrated upregulation of key cardiac genes, including GATA-4, cardiac troponin T, Nkx 2.5 and alpha-myosin heavy chain.

The generation of heart-mimicking tissues can also be achieved based on hydrogels. Zwi-Dantis et al. produced MRI-detecTable 3D collagen hydrogels with aligned human cardiomyocytes from collagen by embedding IONP-loaded cells and transient application of a magnetic field, which resulted in no alteration of normal cardiac function after transplantation onto rat hearts [271]. Another study demonstrated an infection-inhibiting effect of nanoparticles [272]. By incorporating IONPs into bioengineered porous type I collagen patches to repair damaged myocardium, Mahmoudi et al. developed a biodegradable material that could be imaged with MRI and also prevented the growth of Salmonella bacteria in the presence of the embedded NPs. In another work, it was shown that internalization of DMSA-IONPs by cardiac cells into collagen/Matrigel-based 3D engineered cardiac tissues increased biological activity and assembly of gap junctions, enhanced the assembly of electrochemical junctions and decreased adherens junctions and desmosomes [273].

Allginate scaffolds are among hydrogels most commonly tested in tissue engineering of cardiac patches. It has been shown that prevascularization, by the addition of a mixture of prosurvival and angiogenic factors, significantly increases the repair capacity of such transplanted patches [274]. The same group demonstrated that capillary-like networks, even without the addition of angiogenic factors, can be generated by the use of magnetically labeled ECs seeded in macroporous alginate scaffolds through stimulation by an alternating magnetic field [275]. In another study, they demonstrated the generation of functional cardiac patches by combining the use of macroporous alginate scaffolds impregnated with IONPs and seeded with neonatal rat cardiac cells and stimulation by external magnetic fields [276].

In a rat model of chronic myocardial infarction, Blondiaux et al. showed that fibrin patches containing IONPs-loaded human BM-MSCs enhanced myocardial regeneration, and presumably serve as paracrine reservoirs that allow increased release of soluble mediators [277]. In a similar study, cardiopatches of IONP-labeled bone morphogenetic protein 2 (BMP-2)-primed cardiac-committed mouse ESCs were embedded in a biodegradable fibrin matrix and transplanted onto infarcted rat hearts. The results demonstrated efficient cell implantation and improved global and regional cardiac function [278].

A potential treatment of myocardial infarction or severe ischemic diseases by magnetic tissue engineering of multilayered cell sheets was proposed by Ishii et al. [279,280]. In one work, adipose-derived regenerative cells (ARCs) were isolated from wild-type mice, loaded with IONP-containing liposomes (MCLs), and mixed with a diluted ECM precursor. Using a magnet, multilayered cell sheets were formed after 24 h, which, when transplanted onto infarcted mouse myocardium, resulted in significant improvements in systolic function, infarct wall thinning, and angiogenesis.

Scaffoldless multilayered cell sheets of cardiomyocytes (CMs) were prepared by Shimizu et al. using cationic magnetite liposomes-loaded rat CMs by magnetic tissue engineering [281]. Immunofluorescence staining of connexin 43 confirmed the presence of gap junctions, and extracellular potential mapping verified the presence of electrical connections. A similar approach was pursued by Akiyama et al. for magnetic-based production of multilayered cell sheets composed by a mixture of IONP-loaded cardiomyocytes, and ECM precursor [282]. A cylinder placed in the center of the cell culture wells resulted in a homogeneous 3D ring-shaped tissue densely packed with cardiomyocytes, which exhibited contractile properties and was electrically excitable.

Kito et al. generated induced pluripotent stem (iPS) cell layers for therapeutic angiogenesis [283]. For this purpose, mouse IPS cell-derived Flk-1+ cells were loaded with magnetic nanoparticle-containing liposomes (MCLs) and mixed with diluted ECM precursor. Multilayer cell sheets were then generated using an external magnet. Implantation of the cell sheet accelerated revascularization of ischemic hind limbs in nude mice and increased expression of VEGF and bFGF in ischemic tissue.

Mechanical stimulation and physical forces can lead to cell differentiation or conditioning. Chouhan et al. developed a silk fibroin-based and magnetically responsive matrix by incorporating IONPs and seeded it with neonatal rat cardiomyocytes and H9c2 cells [284]. The influence of pulsed magnetic field resulted in significantly increased cell proliferation and higher expression of the connexin 43 gene, indicating the potential stimulation of cultured cardiac cells to develop functional artificial constructs. In addition, the magnetic actuator device also demonstrated the ability to be loaded with drugs and showed a differential drug release profile depending on the stimulation frequency.

Functional tissue engineering of heart valves is a major challenge. To observe cell migration into the developing tissue structure under dynamic conditions, human vascular SMCs, ECs and BM-MSCs were labeled with IONPs and then seeded onto a nonwoven scaffold of a mixture of polyglycolic acid (PGA) and PLA in a hybridization tube [285]. Mechanical conditioning under dynamic flow conditions was performed in a flow chamber of an MRI-compatible bioreactor and enhanced cell migration of SMCs, ECs and BM-MSCs within the scaffold and significantly increased extracellular collagen content.

## 4. Hard and Connective Tissue Regeneration and Engineering

**Figure 4 nanomaterials-11-02337-f004:**
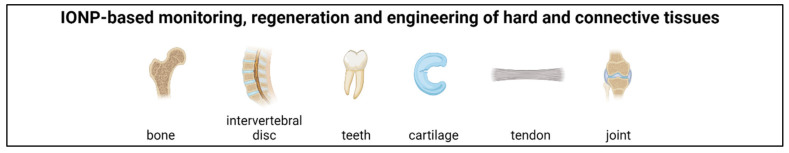
Possible targets for IONP-assisted tissue engineering and regeneration of hard and connective tissues (Created with BioRender.com).

In the following chapter, relevant work in the field of stem cell therapy and tissue engineering for cartilage and bone defects in which IONPs were utilized are described (Figure 4). Consideration will be given to work that uses IONPs as drug carriers or as tools for fabricating composite scaffolds, as well as work that exploits the magnetic properties of IONPs for visual inspection and control of cell- and material-based treatments, for magneto-mechanical induction to regulate cell function and differentiation, or for targeted enrichment of NP-loaded cells.

### 4.1. Cartilage

#### 4.1.1. MRI-Assisted Stem Cell Therapy for Cartilage Regeneration

IONPs represent a commonly used labeling agent that can effectively and rapidly label most cells in vitro without exerting excessive negative interference on cellular functions, such as proliferation or differentiation. This labeling allows the non-invasive in vivo control and monitoring of cells applied for cartilage regeneration or cartilage tissue engineering [286,287]. In this context, Saha et al. investigated the effect of IONPs (ferucarbotran) on chondrogenic differentiation, viability, morphology and proliferation of human BM-MSCs, neonatal and adult chondrocytes [288]. The results indicated that downregulation of chondrogenic genes is time- and cell type-dependent and that ferucarbotran at the concentration used appears to be suitable for noninvasive monitoring of stem cells and mature chondrocytes. Another study showed that the influence of IONPs on stem cell differentiation also depends on the colloidal stability of the nanoparticles before cellular uptake [289]. While stable nanoparticles do not appear to have any effect on osteogenesis or adipogenesis and these exerted a deleterious effect on chondrogenesis only in the presence of high intracellular iron levels, the harmful influence became more pronounced in the presence of nanoparticle aggregates, while adipogenesis and osteogenesis were still unaffected. Jing et al. investigated the fate of IONP-labeled BM-MSCs after injection into the knee joint cavity of rabbit models of cartilage defects by MRI and found that although the injected cells migrated into the synovial fluid at the suprapatellar bursa, the popliteal space site, and the subchondral bone of the femur, they did not actively participate in the repair of articular cartilage defects [290].

#### 4.1.2. Magnetically-Based Targeted Cell Therapy for Cartilage Regeneration

In addition to the possibility of enhancing proliferation, differentiation, and migration of stem cells by mechanical stimulation of an applied external magnetic field, magnetically based enrichment of IONPs or IONP-loaded cells at diseased or defective sites is another promising approach in the field of tissue regeneration [291]. Chiang et al. developed transforming growth factor (TGF)-β1-loaded magnetic gelatin nanocapsules composed of hexanoic anhydride-grafted gelatin and iron oxide nanoparticles to enable combined treatment by magnetically induced stimuli and TGF-β1, as well as to enable magnetic-based enrichment of loaded cells at the site of action [292]. Loading of a chondrogenic ATDC5 cell line with nanocapsules resulted in the magnetically induced upregulation of Col2a1 and, after release of TGF-β1 from degrading nanocapsules, further increased Col2a1 and aggrecan expression and stimulated chondrogenesis. Using an in vitro phantom of a cartilage defect site, Feng et al. demonstrated that SPION-labeled human BM-MSCs can be magnetically directed to the target region to form a three-dimensional (3D) cell sheet structure at the location of the defect [293]. For the treatment of patellar defects, Kobayashi et al. demonstrated that magnetically-labeled MSCs can be enriched after injection into knee joints of rabbit and pig models at the site of the osteochondral defect under the effect of an external magnetic force [294]. The results suggested the potential use of the minimally invasive technique for other cartilage defects caused by osteoarthritis or trauma. In a recent study, autologous BM-MSCs were magnetized with ferucarbotran and injected into the knee joint in patients with a focal articular cartilage defect to which a 1.0 T magnet was pre-attached at an appropriate site [295]. Magnetic targeting demonstrated complete closure of the defects with cartilage-like tissue and significant improvement in the clinical outcomes 48 weeks post treatment.

#### 4.1.3. Tissue-Engineered Cartilage

One of the most widely used scaffold materials, especially for tissue engineering of bone and cartilage, is collagen. Mertens et al. incorporated ultra-small IONPs passively or actively by chemical conjugation during crosslinking into collagen-based scaffolds. The study showed not only a high biocompatibility of the structures towards different cell types, but also that they can be easily, efficiently and stably labeled with ultra-small IONPs. The collagen patches could be precisely monitored by MRI for up to 22 days after subcutaneous implantation into the hind limb of mice [296]. In the study published by Liu et al., MSCs were loaded with N-alkyl-PEI 2k stabilized IONPs and dispersed in a collagen type I hydrogel before the nanocomposite was injected subcutaneously into the flank of mice. Again, the hydrogel could be visualized by MRI for at least 19 days after transplantation [297]. Recently, Huang et al. published a study involving the preparation of magnetic nanocomposite hydrogels from Fe_3_O_4_, polyvinyl alcohol (PVA) and type II collagen, which exhibited good physical properties as well as good swelling behavior and cell compatibility [298]. In another work, Yang et al. fabricated scaffolds of cross-linked collagen/cellulose nanocrystals in which ultra-small IONPs functionalized with the chondroinductive small molecule kartogenin were incorporated [299]. The resulting microenvironment stimulated the growth and differentiation of BM-MSCs and thus the formation of chondrocytes.

Chen et al. demonstrated the noninvasive monitoring of hydrogel degradation by multiparametric MRI during cartilage regeneration using a hydrogel system composed of ultra-small IONPs, cellulose nanocrystal and silk fibroin and confirmed it by histological analysis in a rabbit cartilage defect model [300]. Furthermore, no changes in mechanical hydrogel properties or viability of BMSCs were detected by ultra-small IONP treatment. In the study of Yang et al. a cellulose nanocrystal/dextran hydrogel with embedded ultra-small IONPs was reported which were functionalized with KGN, a non-protein compound that can promote the differentiation of BM-MSCs into chondrocytes [301]. In vitro and in vivo studies showed that KGN indeed recruits endogenous host cells and stimulates BM-MSCs to differentiate into chondrocytes and that the regenerated cartilage tissue closely resembles natural hyaline cartilage. Ramaswamy et al. fabricated two different cartilage constructs composed of polyvinylidene difluoride (PVDF) fibers and poly(ethylene oxide) diacrylate polymer hydrogels, which were both colonized with ferumoxide-labeled chondrocytes derived from the patellar groove and femoral condyle of calves [302]. Using histological methods, they demonstrated that the spatial location of the IONP-loaded cells matched the position detected via MRI, confirming the utility of MRI-based visualization and tracking.

Nedopil et al. developed a protocol for the preparation of matrix-associated stem cell implants from agarose and ferumoxides-loaded adult MSCs for articular cartilage repair and noninvasive in vivo tracking of the therapeutic progress [303]. Another group prepared a magnetically stimulable, three-layer biomimetic cartilage graft using different concentrations of agarose that exhibited elastic and depth-dependent elongation properties and that formed a gradient with sulfated glycosaminoglycan by colonization with bovine chondrocytes [304]. Hou et al. developed cartilage tissue mimetic pellets with excellent structural stability and cytocompatibility consisting of chondrocytes from New Zealand White rabbit articular cartilage, hyaluronic acid-amphiphilic gelatin microcapsules with embedded IONP [305]. Magnetic stimulation enhanced chondrogenesis and synthesis of sulfated glycosaminoglycan (sGAG) and cartilage tissue-specific gene expressions of Col II and SOX9. Preliminary results after transplantation of microcapsules and chondrocytes in an osteoarthritis rabbit model with a pre-implanted magnet in the femoral head, showed that the presence of a magnetic force improved the retention and biofunctionality of the transplanted chondrocytes. Daňková et al. developed a nanofibrous scaffold of PCL and IONP by electrospinning and demonstrated in vitro that the composite improved the viability of MSCs from the bone marrow of miniature pigs, accelerated proliferation, and increased support for differentiation of MSCs compared with IONP-free scaffolds [306].

#### 4.1.4. Drug Supported Cartilage Tissue Engineering

Magnetic stimulation can be used to release growth factors and drugs from stimuli-responsive polymeric systems in a controlled manner to influence the differentiation of cells in a required direction [307]. Fan et al. has developed a magnetic IONP-encapsulated biopolymer nanogel composed of chitosan and heparin via specific nucleobase pairing for vector delivery of BMP-2, which plays an important role in cartilage and bone development [308]. In vitro, the nanogel showed high efficiency in supporting the viability of the osteosarcoma cell line MG-63, especially under a magnetic field. Kim et al. prepared a magnetoresponsive gel of alginate with conjugated heparin and loaded with the TGF-β1 [309]. TGF-β1, which has a heparin-binding domain, was released in a controlled manner by an applied magnetic field, thereby enhancing chondrogenic differentiation of the murine chondrogenic cell line ATDC5. Another study demonstrated the preparation of magnetic scaffolds of silk fibroin and nanoparticles carrying basic fibroblast growth factor (bFGF) by an electrogelation process [310]. In the presence of bFGF-IONPs, the mechanical properties as well as the viability and growth of the osteosarcoma cell line SaOS-2 were improved and showed an inductive effect on collagen synthesis, alkaline phosphatase activity and total protein synthesis.

#### 4.1.5. Scaffold-Free Cartilage Tissue Engineering

In addition to fabricating composites based on cells and biomaterials, there are approaches to generate tissues using only cells without supporting materials. Son et al. loaded human BM-MSCs with IONP from Magnetospirillum sp. AMB-1 and exposed them to a static magnetic field after pelleting [311]. Magnetic stimulation increased the content of collagen and sulfated glycosaminoglycan (sGAG), the main components of cartilage tissue-specific ECM, and improved chondrogenic cell differentiation. Murata et al. demonstrated the feasibility of homogeneous labeling and visualization of human iPS cell-derived 3D cartilage tissue [312]. After dissociation of cartilage tissue and labeling with gelatin nanospheres containing three types of quantum dots (QD) with different fluorescence wavelengths and IONPs, the labeled cells were processed into 3D pellets or cell sheets and could be fluorescently visualized over 4 weeks.

### 4.2. Bone Regeneration

**Figure 5 nanomaterials-11-02337-f005:**
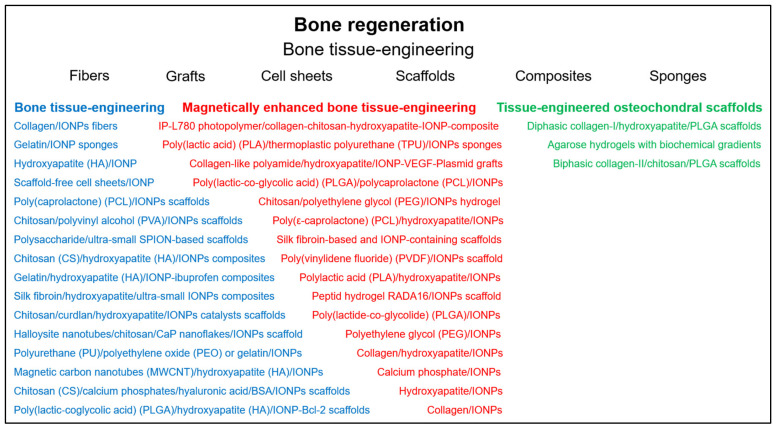
Stem cell therapy and tissue-engineering for bone regeneration. Possible strategies for bone regeneration and examples of materials and composites from studies reviewed below.

Reconstruction of bone tissue is an important goal in regenerative medicine. Promising approaches include the controlled differentiation of stem cells and the use of different materials to generate bone scaffolds (Figure 5). The use of IONPs has been demonstrated not only to allow the visualization of the therapeutic process by MRI imaging, but also to promote cell differentiation and, through the application of magnetic fields, to enhance osteogenesis and even to specifically deliver labeled cells or drugs to the target site [313,314,315].

#### 4.2.1. Stem Cell Therapy for Bone Regeneration

As in other cell-based therapies, stem cell therapies for bone regeneration can be non-invasively monitored and tracked after pre-loading of the administered cells with IONPs. For example, Kérourédan et al. used a laser-assisted in situ bioprinting method to print viable human stem cells from apical papilla onto a mouse calvarial defect. Printed patterns of cells with superparamagnetic microspheres demonstrated the feasibility of noninvasive cell tracking by MRI [316].

Apart from the suitability for imaging, the tissue repair-stimulating effect of IONPs has been known for a while, but the exact underlying mechanisms are still unclear. Using gene microarray assays and bioinformatics analyses, Wang et al. have shown that gene expression of human BM-MSCs is strongly regulated after treatment with IONPs and that the classical mitogen-activated protein kinase (MAPK) signaling pathway is activated, thereby regulating downstream genes and promoting osteogenic differentiation [317]. Schulze et al. investigated the effect of amino-polyvinyl alcohol (PVA)-coated IONPs on BM-MSCs after systemic injection into rat veins and found an accumulation of the particles in the bone marrow and an increase in the metabolic activity and migration rate of BMSCs [318]. Thus, the particles have high potential MRI imaging of bone marrow and may be of interest for future applications in regenerative medicine.

Another possibility to positively influence cell differentiation is mechanotransduction. The application leads to a conversion of an external force acting on a cell into internal biochemical signaling pathways and affects cell differentiation, proliferation and ECM composition, among others, and can be used in regenerative medicine for tissue-forming approaches [319,320,321]. Hu et al. developed a Magnetic Force Bioreactor for ex vivo induced bone formation by magneto–mechanical stimulation, capable of activating specific cell surface receptors, such as platelet-derived growth factor receptor α (PDGFRα), using IONPs [322]. Thus, mechanical stimulation by magnetic labeling could positively affect osteogenesis and mineralization of MSCs. Rotherham et al. activated Wnt signaling in human MSCs by IONPs functionalized with UM206, a synthetic peptide and ligand for the Wnt receptor Frizzled [323]. Injection and magnetic stimulation of UM206-IONP-labeled MSCs into ex vivo chick femurs resulted in enhanced osteogenesis and mineralization.

#### 4.2.2. Bone Tissue-Engineering

The addition of IONPs to scaffolds can promote protein adsorption and the formation of a protein corona, which exerts a positive influence on tissue regeneration. Zhu et al. have provided insight into the underlying molecular mechanisms of the osteoinductive effect of IONPs in magnetic hydroxyapatite (HA) scaffolds and demonstrated that functional proteins enriched in the corona efficiently activate the MAPK/ERK signaling pathway, ultimately leading to the promotion of cell proliferation [324,325]. In an in vivo bone repair model, they showed that the dynamic protein corona formed on the scaffolds promotes the activation of acute inflammatory responses and leads to the recruitment of immune cells, remodeling of the ECM, and acceleration of bone healing.

HA is one of the most frequently used substances for bone tissue engineering, mostly as part of biocomposites. Heidari et al. examined the properties of pure chitosan, chitosan /HA, chitosan /HA/magnetite and chitosan/magnetite, materials used for bone tissue engineering, and showed that all composites are cytocompatible and that the addition of HA and magnetite to the chitosan matrix can significantly improve the mechanical properties of pure chitosan [326]. In a study by Russo et al., implantation of a porous HA/magnetite scaffold in a rabbit model of a critical femoral defect was shown to induce and support bone tissue formation with no short-term adverse effects on biocompatibility and bone formation ability compared to IONP-free HA, the current gold standard in the treatment of critical bone defects [327]. Sahmani et al. produced bio mimicking scaffolds with drug delivery capability [328]. The porous scaffolds, which can potentially be used for biological as well as hyperthermal applications, were prepared from HA and IONPs and loaded with gelatin into which ibuprofen, a non-steroidal and non-inflammatory substance, was incorporated. In another study, Przekora et al. investigated the effects of oxygen and nitrogen species on proliferation and osteogenic differentiation of human ASCs [329]. They embedded FexOy/NPs catalysts in the polysaccharide matrix of chitosan/curdlan/HA and colonized the scaffold with ADSCs. Short-term exposure of ADSCs to nitrogen plasma accelerated stem cell proliferation without negatively affecting osteogenic differentiation. Liu et al. demonstrated the feasibility of non-invasive in vivo monitoring of the biodegradation of silk fibroin/HA/ultra-small IONPs bone scaffolds implanted subcutaneously into the back of nude mice after loading with BM-MSCs using quantitative MRI [330]. In addition to promoting cell adhesion and cell growth and facilitating osteogenesis, resorption of the scaffolds occurred with concomitant bone formation. In the study of Pistone et al., a biocompatible nanocomposite system of HA, IONP and magnetic multi-walled carbon nanotubes was developed for controlled drug delivery and represents a potential multimodal platform that has the potential to be used for bone tissue engineering [331]. Finally, Brett et al. fabricated a HA-PLGA scaffold containing IONPs functionalized with a minicircle plasmid to overexpress B-cell lymphoma 2 (Bcl-2) to inhibit apoptosis in implanted cells [332]. The constructs were implanted into a mouse calvarial defect model and seeded with adipose-derived stromal cells. After in vivo magnetofection, a significantly higher rate of bone regeneration was observed in the center of the scaffold after 8 weeks compared with control groups.

In addition to the widespread use of HA-containing biocomposites in bone tissue engineering, several other studies have demonstrated the functionality of biocomposites without the involvement of HA. Cojocaru et al. developed biodegradable composites with a macroporous structure containing chitosan, calcium phosphates, hyaluronic acid, and bovine serum albumin and IONPs that showed good biocompatibility toward osteoblast cells [333]. To enhance osteogenesis of human ASCs, Lee et al. introduced a nanoscaffold of halloysite nanotubes modified with IONPs, chitosan and 2-D calcium phosphate nanoflakes. Through the synergistic osteoconduction of IONPs, chitosan and calcium phosphate, the nanoscaffolds induced significantly enhanced osteogenesis of well-differentiated osteoblasts [334]. Lalande et al. demonstrated the feasibility of in vivo monitoring of human ASCs pre-loaded with ultra-small IONP-labeled agents and seeded into a porous polysaccharide-based 3D scaffold [335]. After subcutaneous injection into nude mice, the human ASCs remained detectable by MRI for up to 1 month and migrated out of the scaffold and populated the surrounding area.

Due to their biocompatibility, gelatin-containing scaffolds were also utilized for tissue engineering. In a study by Wang et al. biodegradable bone scaffolds were fabricated from shape memory PU, IONPs and polyethylene oxide (PEO) or gelatin by 3D printing [336]. After colonization with human MSCs, both scaffolds promoted osteogenesis through the gradual release of IONPs with the PU-gelatin scaffolds exhibiting better cell viability and the PU-PEO scaffolds exhibiting better shape memory properties. In another study, Hu et al. fabricated an MRI-visualizable IONP-loaded gelatin sponge for bone regeneration and demonstrated a strong IONP-induced active osteogenesis after implantation into the incisor sockets of rats [337].

Apart from the above-mentioned possibilities for tissue engineering, there are also other innovative methods including electrospinning. Wei et al. developed a biodegradable magnetic nanofiber material of IONPs/chitosan/PVA, which exhibited improved cell adhesion and proliferation over MG63 human osteoblast-like cells compared to cell culture plates [338]. The biological and physicochemical properties of silk fibroin can be improved by blending or crosslinking with other biopolymers, such as collagen, chitosan, alginate and hyaluronic acid, and thus often serve as materials for tissue engineering applications [339]. Lalegül-Ülker et al. proposed elektrospinned silk fibroin with embedded IONPs as potentially suitable for the generation of magnetically responsive cytocompatible polymer composites [340]. Singh et al. also used electrospinning for the fabrication of PCL and IONPs nanofiber scaffolds, which, due to the incorporated IONPs, exhibited enhanced hydrophilicity and mechanical tensile properties, increased nanofiber degradation, and improved cell adhesion and osteogenesis of osteoblasts [341]. Finally, in vivo studies in rats confirmed the bone regeneration ability and tissue compatibility of the PCL-IONP scaffolds.

#### 4.2.3. Magnetic Force-Based Bone Tissue-Engineering

Zhuang et al. presented a magnetically assisted electrochemical technique to deposit type I collagen nanofibers onto a substrate. The embedded IONPs and an external magnetic field resulted in aligned collagen coatings that promoted the growth of BM-MSCs in the form of elongated morphology, which strongly promoted cellular osteogenesis [342].

In contrast to the aforementioned biocomposites, cell sheet techniques offer the possibility of producing a tissue without the use of supporting structures. Silva et al. produced hierarchical vascularized heterotypic 3D cell constructs from IONPs-loaded human umbilical vein ECs (HUVECs) and ASCs by magnetic force-based cell sheet engineering [343]. The heterotypic cell sheets showed increased alkaline phosphatase activity, matrix mineralization, osteopontin and osteocalcin levels, and induced osteogenesis and blood vessel recruitment in vivo.

#### 4.2.4. Magnetic Force-Enhanced Stimulation of Engineered Tissues

Magnetic scaffolds have the potential to enhance bone tissue formation by plain magnetic stimulation. Russo et al. addressed the preparation of PCL matrix and IONPs and the effect of time-dependent magnetic field on adhesion, differentiation and proliferation of human MSCs [344]. The results showed prolonged cell differentiation and increased ERK phosphorylation levels, thus activation of the MAPK signaling pathway. Xia et al. investigated the in vitro and in vivo osteoinductivity of IONP-incorporated calcium phosphate cement (IONP-CPC) scaffold on human dental pulp stem cells (DPSCs) and showed that IONP-CPCs had better cell spreading, and greater bone mineral synthesis and ALP activity, than CPC controls and that cell differentiation was likely driven via the WNT signaling pathway [345,346]. They also demonstrated that the differentiation of stem cells was enhanced by the magnetic field and increased active osteogenesis in mandibular defects of rats, compared to IONP-free scaffolds [347]. Zeng et al. fabricated magnetic biomimetic scaffolds made of HA by immersing HA scaffolds in IONP solutions [348]. After cell colonization, the rat osteosarcoma cell line ROS 17/2.8 and the murine osteoblast precursor cell line MC3T3-E1 experienced stimulation of cell proliferation and differentiation by the intrinsic magnetic field, which was further enhanced by an applied external magnetic field. Similarly, the study of Tanasa showed that the presence of applied magnetic field in scaffolds prepared from silk fibroin, poly(2-hydroxyethyl methacrylate) and IONPs increased the proliferation of murine 3T3-E1 pre-osteoblasts and positively affected the osteogenic differentiation capacity [349].

Many studies on bone tissue engineering are based on the preparation of hydrogels with embedded IONPs. These biocomposites have demonstrated a strong impact on cell differentiation under magnetic fields. Filippi et al. prepared magnetizable nanocomposite hydrogels by incorporating IONPs into PEG-based hydrogels containing cells derived from human adipose tissue [350]. Under a static magnetic field, cells showed an increase in metabolic activity, expression of osteogenic markers, and deposition of mineralized matrix and, after subcutaneous implantation in mice, denser, more mineralized, and more rapidly vascularized tissue than in the control group. In a study by Aldebs et al., biomimetic hydrogels were prepared from the self-assembling peptide RADA16, which consists of regular repeats of alternating ionic hydrophilic and hydrophobic amino acids, IONPs, and human ASCs [351]. Direct stimulation by extremely low frequency pulsed electromagnetic fields resulted in early differentiation of human ASCs into an osteoblastic phenotype. Cau et al. prepared a chitosan/ PEG hydrogel with embedded IONPs and showed that under an alternative magnetic field (AMF) and elevated temperature at 43 °C, the main temperature used for hyperthermia treatment, the osteogenic differentiation capacity of MSCs was significantly increased compared to direct heat treatment [352]. Yuan et al. developed a bone model composed of plastic compressed collagen with embedded IONPs and osteoblast cells (MG-63) and showed that in the presence of static magnetic fields, the composite stimulated alkaline phosphatase production and mineralization of the cells by affecting matrix/cell interactions and promoting the expression of BMP-2 and BMP-4, runt-related transcription factor 2 (Runx2) and osteonectin [353]. The study by Hao et al. used PLGA and IONPs composites to investigate the synergistic effects of magnetic nanocomposites and of external magnetic fields on osteogenic differentiation of osteoblasts [354]. The results demonstrated a significantly enhanced cell attachment and differentiation through increased alkaline phosphatase (ALP) activity, upregulation of the expression of bone-associated genes (ALP, OCN, and BMP2) and increased mineralized nodule formation.

In addition to the usual techniques for producing scaffolds, such as blending for hydrogel formation, some other techniques are used as well. By electrospinning IONPs, HA-NPs, and PLA, Meng et al. fabricated magnetically responsive nanofibrous scaffolds that accelerated the formation of new bone tissue under an external static magnetic field after transplantation into a rabbit model of lumbar transverse defects by stimulating osteoblast cells proliferation and secretion of new ECM [355]. Using a combination of electrospinning and layer-by-layer assembly of IONPs, Chen et al. fabricated magnetic PLGA/PCL scaffolds that, through their surface properties and especially via their magnetic responsivity, significantly enhanced osteogenic differentiation compared with scaffolds containing gold nanoparticles [356].

By a solvent-casting method, Fernandes et al. fabricated a magnetoactive scaffold of three different fiber diameters with different pore sizes using a piezoelectric polymer, poly(vinylidene fluoride) (PVDF) and CoFe_2_O_4_ NPs, which stimulated the proliferation of preosteoblasts by applied magnetic fields [357]. Marycz prepared IONP-doped sponges of thermoplastic PU and PLA polymer using the solvent casting technique and demonstrated increased expression of osteopontin and collagen type I and decreased expression of BMP-2 and subsequently enhanced osteogenic differentiation of ASCs under a static magnetic field [358]. In a study from Aliramaji et al., silk fibroin/chitosan-based magnetic scaffolds with different amounts of IONPs were prepared by freeze-casting method and demonstrated that under static magnetic fields, the IONP can enhance cell adhesion and the number of living cells [359]. Finally, by laser writing via two-photon polymerization of IP-L780 photopolymer and coating with collagen-chitosan-HA-IONP composite, Paun et al. produced a biomimetic structure that accelerates bone regeneration, especially under static magnetic stimulation, in vitro, by enhancing the differentiation of human osteoblast-like MG-63 cells [360].

#### 4.2.5. Magnetic Force-Based Attraction of Agents to Engineered Tissues

Magnetic scaffolds are suitable for transporting magnetically labelled soluble factors such as growth factors, hormones and polypeptides or cells directly to the site of implantation. A method for in vivo loading of scaffolds with bio-agents was demonstrated by Bock et al. [361]. By immersing commercially available scaffolds composed of HA and collagen in aqueous IONP solutions, magnetizable scaffolds were created with the ability to attract growth factors, stem cells or other bio-agents bound to magnetic particles by magnetic propulsion. In another study, Panseri et al. compared the in vivo biocompatibility and osteointegrative properties of magnetic hydroxyapatite/collagen scaffolds prepared either by simultaneous nucleation of apatite and IONPs on self-assembling collagen fibrils or by impregnation in ferrofluid solution after implantation in rabbits [362]. Both scaffolds showed no inflammatory response, good bone healing rates and appear promising in attracting functionalized IONPs or IONP-loaded cells after implantation by a driving magnetic force. Luo et al. enabled the internal vascularization of scaffolds prepared of hydroxyapatite and collagen-like polyamide by loading magnetic plasmid gene microspheres to repair large bone defects [363]. Magnetic field-released plasmid genes led to transfection of surrounding cells and protein expression of vascular endothelial growth factor (VEGF), and finally to angiogenesis and osteogenesis of the scaffold in a rabbit model of a large segmental radius bone defect.

#### 4.2.6. Tissue-Engineered Osteochondral Scaffolds

In the meantime, techniques have been invented that allow a differentiated development of tissues, e.g., the simultaneous locally separated formation of bone and cartilage tissue. Su et al. produced biphasic type II collagen-chitosan/PLGA scaffolds with IONP-labeled rabbit chondrocytes, whose cellular distribution and proliferation within the scaffold could be magnetically controlled and also exhibited increased gene expression of type II collagen and aggrecan [364]. In another study, Huang et al. fabricated a diphasic magnetic nanocomposite scaffold composed of collagen-I, nanohydroxyapatite, PLGA and IONPs seeded with BM-MSCs) [365]. The scaffold exhibited good cell compatibility along with good mechanical properties and showed pore size and porosity similar to the physiological structure of normal articular cartilage and subchondral bone. Li et al. used magnetic fields to guide glycosylated IONPs loaded with BMP-2 to establish biochemical gradients in agarose hydrogels, pre-laden with human MSCs to generate osteochondral tissue constructs that exhibited a clear mineral transition from bone to cartilage [366].

### 4.3. Intervertebral Disc and Joint Repair

Saldanha et al. demonstrated that ferumoxides-labeled MSCs can be detected in vitro in natural and synthetic polymers as well as distinguished in vivo from the native tissue environment in rat intervertebral disc degeneration by MRI, thus appearing potentially suitable for longitudinal in vivo tracking of stem cell-based disc regeneration [367]. Multipotent MSCs are commonly used in cellular therapy for joint repair. To track IONP-labeled MSCs injected into joints with osteochondral defects in sheep, Kaggie et al. used ultrafast MRI using a 3D cone acquisition trajectory, which provided excellent anatomic details [368]. Together with histological staining, it was shown that IONP-loaded cells aggregated at the injection site and did not migrate to the defect site, suggesting that if MSCs are indeed involved in repair, the mechanism of action is in the recruitment of secondary cells.

### 4.4. Tendon

Musculoskeletal tissue injuries such as tendon and ligament pathologies are common in the elderly and especially in athletes, with traumatic rupture of the anterior cruciate ligament and Achilles tendinopathy being the most common [369]. Scharf et al. established an IONP-based cell tracking method in a sheep model of tendinitis [370]. Labeling of ovine BM-MSCs was detectable by MRI for up to 7 days, which was confirmed by correlative histology. The study demonstrates the feasibility of evaluating the location, distribution, and disappearance rate of cells in the musculoskeletal system of large animals in a time-dependent manner.

The feasibility of IONP-based cell tracking was also demonstrated by Kremen et al. [371]. They used genetically modified IONP-loaded and luciferase (Luc+) expressing murine mesenchymal progenitor cells (C3H10T1/2 MPCs) for implantation into nude rats with surgically created Achilles’ tendon defects. The IONPs allowed MRI-based monitoring of the grafts and did not affect viability of the MPCs, as demonstrated by quantitative bioluminescence imaging.

The use of decellularized tendon tissue as a basis for tendon tissue engineering represents a very promising therapeutic approach. Ideally, in addition to the required biocompatibility, scaffolds should approximate natural biomechanical properties as closely as possible. Therefore, decellularization of donor tissue offers the possibility to obtain hypoimmunogenic scaffolds with a natural ECM structure that supports subsequent repopulation with cells. Burk et al. has shown that decellularization of horse tendons by repeated freeze-thaw cycles, especially in combination with Triton X-100, was cytocompatible with IONP-loaded MSCs and showed no morphological changes of the ECM [372].

Another study follows the approach of generating magnetic polymer scaffolds [373]. Aligned fibrous magnetic scaffolds were fabricated from a blend of starch and PCL by rapid prototyping through incorporation of IONPs (Micromod) into SPCL powder. Seeded ASCs underwent tenogenic differentiation under magneto-stimulation conditions and synthesized a collagen type I and tenascin C rich matrix. The scaffolds revealed good biocompatibility and integration with surrounding tissues after subcutaneous implantation in rats, indicating the potential utilization of this approach for tendon regeneration. In a later study, the same group developed magnetically responsive, scaffold-free and ECM-rich patches using a magnetic cell layer technology (magCSs) for tendon regeneration [374]. The magCSs patches, for which tenomodulin-positive (TNMD+) subpopulation of human ASCs was used, may be a promising approach to stimulate endogenous regenerative mechanisms.

### 4.5. Teeth

Interest in regenerative methods in dentistry is growing steadily. There are now a variety of approaches in tissue engineering for almost all anatomical areas of the tooth, in particular the enamel, dentin and pulp [375]. Anastasiou et al. demonstrated the exogenous mineralization of hard tissues based on the use of femtosecond pulsed lasers and fluorapatite powder mixed with IONPs [376]. The photothermal process triggered the sintering and densification of the surrounding calcium phosphate crystals, forming a new layer bonded to the underlying surface of the natural enamel. The material produced in this way was more acid-resistant than natural enamel and its hardness and modulus of elasticity made it significantly better adapted to enamel than the restorative materials currently used in clinical dentistry.

Li et al. developed a magnetic adhesive composed of dimethylaminohexadecyl methacrylate, amorphous calcium phosphate NPs and magnetic NPs that exhibited significantly higher dentin adhesion than the commercial control and reduced S. mutans biofilm colony-forming units by 4 logs [377]. Thus, the material could improve the connection between tooth and resin and suppress secondary caries at the restoration edges.

In a recent paper, a multifunctional periodontal hybrid scaffold was developed that mimics the various compositional and microstructural features of alveolar bone, periodontal ligament and cementum [378]. The scaffold exhibited good cytocompatibility and cell viability and could promote osteogenesis through the bioactive superparamagnetic apatite phase and be activated by remote magnetic signals. In another study, human DPSCs were seeded on calcium phosphate cementum scaffolds with incorporated IONPs [379]. Incorporation of IONP into the scaffold increased osteogenic differentiation and bone matrix mineral synthesis of the cells, indicating a potential approach for improved dental as well as craniofacial and orthopedic bone regeneration. Zhang et al. was dedicated to the development of a biomaterial for dental pulp tissue regeneration and repair with the possibility of noninvasive imaging [380]. An ultra-small IONP-labeled hydroxyapatite/silk fibroin scaffold loaded with DPSCs was implanted with a tooth fragment into the subcutaneous space under the nude mice. Expression of dentin acidic matrix phosphoprotein 1 and dentin sialophosphoprotein indicated odontoblast-like cell differentiation, and optical imaging confirmed good revascularization and mineralization. At about the same time, Zare et al. demonstrated that dental pulp stem cells can be loaded with dextran-coated IONPs without significant reduction in viability, proliferation and differentiation properties, demonstrating the suitability of the MRI-based method for monitoring labeled cells [381].

## 5. PNS and CNS Regeneration

Stem cells and progenitor cells, as well as differentiated cells such as adult neurons, Schwann cells and astrocytes, have been used to treat many different pathologies of the central and peripheral nervous system, including various neurodegenerative diseases such as Alzheimer’s disease (AD), Parkinson’s disease (PD), Huntington’s disease (HD), ischemic stroke, amyotrophic lateral sclerosis (ALS), spinal cord injury and multiple sclerosis (MS) [382,383,384,385].

The use of nanotechnology in diagnostics but also in drug delivery and tissue engineering could significantly improve the treatment of patients with injured neuronal tissue [386,387,388,389,390]. Hence, IONP-labelling of stem cells or other cells, such as astrocytes and microglia can be used to track and monitor transplanted cells after implantation, e.g., by a noninvasive imaging modality such as MRI, magnetic particle imaging, positron emission tomography, and multiple photon microscopy [391,392,393,394,395,396,397,398,399,400,401,402]. Moreover, IONPs and IONP-loaded cells allow the delivery of therapeutic biomolecules, such as neurotrophic factors, drugs, proteins, DNA and siRNA by specific NP functionalization [403,404,405,406,407]. Functionalized IONPs and IONP-loaded cells can be effectively enriched at the injury site by external magnet fields to efficiently promote neuronal repair or guide axonal growth [408,409,410,411,412,413,414,415]. Finally, the use of scaffolds made of various nanomaterials can serve as structural support, induce or mimic the formation of an ECM, inhibit glial differentiation, promote neuronal growth and control hemostasis. In this context, IONPs can be used as part of the biocomposite for diagnostic purposes, to magnetically induce a specific surface topology or neurite outgrowth or to further improve cellular differentiation [416,417,418,419,420].

**Figure 6 nanomaterials-11-02337-f006:**
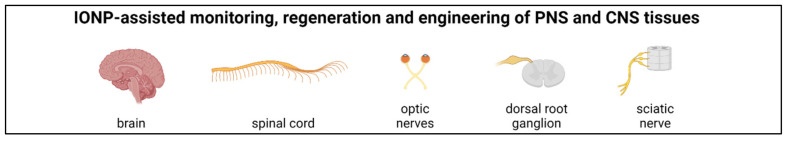
Possible targets for IONP-assisted tissue engineering and regeneration of tissues within the peripheral and central nervous system (Created with BioRender.com).

In the following, we highlight other interesting approaches in the treatment of specific PNS and CNS injuries by stem cell therapy or by the use of nanomaterials as tissue engineering scaffolds. Possible targets for IONP-assisted tissue engineering and regeneration of tissues within PNS and CNS are depicted in Figure 6.

### 5.1. PNS

#### 5.1.1. Dorsal Root Ganglia

Sensory neurons of the dorsal root ganglia (DRG) transmit information from stimuli that trigger feelings of touch, pain, muscle tension and temperature, among others, from the body to the CNS. There a variety of diseases and disorders that directly and indirectly lead to degeneration or dysfunctionality of sensory neurons. In addition to hereditary diseases such as Charcot Marie Tooth Disease and Friedreich’s Ataxia, as well as autoimmune diseases and toxic substances such as alcohol, DRG neurons can be severely damaged by physical trauma [421]. Meanwhile reports are increasing in which IONPs were used to support the regeneration of injured neurons.

For example, in one study, the neurotrophic factors including nerve growth factor (βNGF), bFGF-2, and glial cell-derived neurotrophic factor (GDNF) were coupled to IONP to increase the very short half-life of these compounds. Liu et al. showed that nano-hydroxyapatite (n-HA)-coated IONPs could potentially be used for nerve regeneration by effectively increasing the viability of primary cultured DRG neurons and promoting axonal elongation through activation of the Netrin-1 signaling pathway [422].

The combination of IONPs with hydrogels are another promising approach for the regeneration of DRGs. In NVR-Gel, which is mainly composed of hyaluronic acid and laminin, the functionalized NPs were able to enhance early nerve fiber sprouting compared to free factors in organotypic DRG cultures and significantly accelerate the progression of myelin formation [423]. The study by Assunção-Silva demonstrated the use of synthetic Gly-Arg-Gly-Asp-Ser peptide-modified biodegradable gellan gum-based hydrogels enriched with glial derived neurotrophic factor (GDNF)-coated IONPs to treat severe peripheral nerve injury [424]. The hydrogels supported the attachment and growth of ASCs, enhanced neurite outgrowth from DRG, and, in the presence of GDNF, IONPs alone or in combination with ASCs, significantly increased neurite growth from DRGs.

Other studies were based on the use of electrospun fibers. Zuidema et al. controlled the preferential DRG neurite outgrowth along aligned electrospun PLA microfibers in the presence of nerve growth factor (NGF)-releasing IONPs [425]. Similarly, Johnson et al. fabricated magnetic electrospun fiber scaffolds composed of PLA and oleic acid-coated IONPs [426]. After injection into a collagen or fibrinogen hydrogel solution supplemented with Matrigel and magnetic alignment, the scaffolds provided internal directional guidance to neurites growing from dorsal root ganglion explants and improved the neurite growth.

#### 5.1.2. Sciatic Nerves

Autologous nerve transplantation is an accepted therapy for peripheral nerve repair. Since, among other things, available donor nerves are scarce, there are promising approaches for replacement with biomaterials or biomaterials in combination with cells.

Lacko et al. presented the preparation of aligned tubular hydrogel scaffolds of glycidyl methacrylate hyaluronic acid and collagen I by magnetic templating of dissolvable magnetic alginate microparticles and their subsequent dissolution [427]. The tubular microstructures were able to guide axon extension of dorsal root ganglia, and a pilot study on a 10 mm rat sciatic nerve model showed qualitatively improved axon regeneration compared to non-templated controls.

Liu et al. developed a Schwann cell-loaded magnetic nanocomposite scaffold composed of a biodegradable chitosan-glycerophosphate polymer and IONPs [428]. When inserted into a 15-mm sciatic nerve gap in rats and in the presence of a magnetic field, the scaffold improved Schwann cell viability and promoted nerve regeneration.

Tseng and Hsu investigated the effect of MSC-derived spheroids compared with single cells on the regeneration of transected rat sciatic nerves [429]. They demonstrated that the spheroids have greater differentiation capacity than single cells and accelerated the functional recovery of rats in which the sciatic nerve was transected to a length of 10 mm and bridged by a microporous PLA nerve guide conduit. In addition, IONP-labeled spheroids could be visualized by MRI, whereas the use of brain-derived neurotrophic factor (BDNF)-transfected spheroids resulted in the shortest gap-bridging time, the largest regenerated nerve and the thickest myelin sheath.

### 5.2. CNS

Nanotechnology, in combination with stem cells, offers great potential in the treatment of brain and spinal cord injuries [430,431]. To treat nerve tissue injuries of the CNS, current research involves a variety of different aspects and techniques of cell therapy and tissue engineering. To monitor therapy progress and functional recovery or guide stem cell injection to the injury site, cells or applied biomaterials can be labeled with IONPs and thereby be monitored by several imaging modalities, such as MRI or microwave and optical imaging, among others [432,433,434,435,436,437,438,439,440,441]. The use of biomaterials can promote regeneration by providing structuring fiber scaffolds or ECM-like matrices to regenerate axons [442]. Moreover, regenerative capacity can be enhanced by the use of IONPs. However, even if the NPs appear to be innocuous under normal conditions, the specific NPs needs to be carefully selected as they might induce adverse reactions [443,444]. Nevertheless, IONPs have been shown to enhance neuroregeneration, for example, by their potential to positively influence neuromodulation, to dictate growth direction in response to an external magnetic field, or to enhance regeneration by coupling with growth-promoting agents [445,446].

#### 5.2.1. Optic Nerves

Optic nerve diseases, such as diabetic retinopathy and glaucoma, are the most common causes of blindness [447]. Vision loss occurs when optic nerve injury, such as crushing or transection, leads to axon degeneration and retinal ganglion cell death. There are currently few promising neuroprotective strategies that could promote neuronal survival and protect against vision loss and retinal cell death. These include the administration of various drugs and rehabilitative methods such as exercise and electrical stimulation therapies but also gene and cell therapies [447,448]. Meanwhile, applications have been suggested in which NPs are used as vehicles for the delivery of genes, drugs, and trophic factors, among others [449,450]. However, reports about the usage IONPs are still very rare.

In one study, rat whole-animal MRI and fluorescence analyses demonstrated that IONP-labeled polymeric nanospheres remain at the injury site after injection and also partially penetrate injured axons and are transported to the parent somata, thus offering the possibility of delivering therapeutic agents to the injury site and somata of injured CNS neurons [451]. The work of Giannaccini et al. demonstrated in a model of oxidative stress-induced retinal ganglion cell (RGC) loss that IONP-coupled neutrophins, such as NGF and BDNF, completely prevented RGC loss, in contrast to the administration of the free drugs [452]. In a rat model of optic nerve crush, injection of IONP-loaded MSCs into the vitreous body was shown to exert a neuroprotective effect on RGCs and stimulate axon regeneration [453]. Finally, Pita-Thomas et al. used IONPs functionalized with antibodies against retinal ganglion cell (RGC) membrane molecules and an external magnetic field to induce a directed mechanical force on growth cone filipodia and thereby filopodia elongation and growth [454]. However, future experiments will be needed to determine whether this approach can be optimized to induce axonal growth.

#### 5.2.2. Spinal Cord

Spinal cord injuries often lead to pain and permanent loss of sensory and motor functions. Currently, there is still no established therapy to restore the lost functions in patients, although research into possible solutions has been ongoing for a long time. Despite reports that transplantation of IONP-labeled BM-MSCs did not result in clinical improvement in neurologic function [455], there are many promising approaches and developments in cell therapy and tissue engineering for the treatment of spinal cord injury that have already been tested in animal models and patients [456,457]. Stem cell transplantation is currently considered the most promising therapeutic approach for spinal cord injury. Labeling with IONP offers a very beneficial way to evaluate the fate of transplanted or injected cells and the success of therapy [458,459,460,461,462,463,464,465,466,467,468,469,470,471,472].

##### Spinal Cord Regeneration by IONP-Enhanced Cell Therapy

Besides the very advantageous possibility to observe IONP-loaded cells during therapy by imaging methods, cells and their regenerative capacity can also be positively affected by uptake of IONPs—either by magnetically controlled targeting or/and by specific functionalization.

In a study by Cho et al., a pulsed electromagnetic field (PEMF) directed IONP-loaded human BM-MSCs to the vicinity of the spinal cord-injured site in rats, resulting in improved behavioral tests [473]. Other studies confirmed a rapid targeting of IONP-loaded MSCs to rat spinal cord lesions in presence of external magnetic fields and the benefits for neuronal regeneration and neuropathic pain treatment [474,475,476]. Another group developed a magnetic targeting system using an implanted magnet to guide IONPs-labelled BM-MSCs to the injured site in the spinal cord of rats after subarachnoid injection [477,478]. Similarly, Vaněček et al. increased the targeting efficiency of transplanted IONP-labeled MSCs near the lesion site in rat spinal cord after intrathecal injection and the use of an implanted plate-shaped permanent magnet [479].

Several other studies demonstrated positive effects of cell therapy with incorporated functionalized IONPs. Choi et al. demonstrated that human ASCs loaded with core-shell particles made of IONPs with a ZnO envelope enhanced the regenerative activity and effectively alleviated neuropathic pain in induced traumatic spinal cord injury mouse models [480]. However, not only the regenerative capacity of stem cells can be enhanced by loading with IONPs, but also by other cells such as glial cells. Glia cells can alleviate the formation of scars followed by spinal cord injuries and enhance axonal growth by releasing bioactive substances and potentially create a glia bridge in the CNS environment to guide regenerated axons to their distal destination. However, the efficacy of Schwann cell (SC) transplantation is limited by the poor migratory ability of SCs into the astrocyte-rich CNS environment. To enhance transplantation efficiency, IONP-loaded SCs can be guided into the astrocyte-rich region by a directional magnetic field [481]. In addition, transfection of SCs with chondroitinase ABC (ChABC)-polyethylenimine-functionalized IONPs can overexpress ChABC and induce the removal of chondroitin sulfate proteoglycans, thereby inhibiting scar formation [482]. In another study, SCs were initially magnetofected with polysialyltransferase-functionalized IONPs to induce the overexpression of polysialylation of neural cell adhesion molecule (PSA-NCAM) to enhance the migratory ability [483]. The migration direction of the SCs could then be controlled by an applied magnetic field, causing the cells to migrate efficiently into the astrocyte domain.

The transplantation of olfactory ensheathing cells (OEC), a type of glia which ensheat axons of the olfactory receptor neurons, have shown promising results in promoting the regeneration of CNS axons after transplantation into the injured CNS [484]. IONP-labelling was shown not to affect cell proliferation, migration and viability and it has been demonstrated that IONP-labelled OECs can be controlled by an external magnetic field and integrate in organotypic slices of spinal cord and peripheral nerves [485,486]. In another work, however, the migratory and regenerative capacity of OEC has been shown to be inefficient when challenged with the glial scar of a transected spinal cord [487]. However, it has been shown that genetic modification of OECs could enhance their therapeutic potential by secreting neurotherapeutic factors. Delaney et al. showed that magnetic particles combined with magnetic fields and DNA minicircle vectors can safely bioengineer OECs to secrete important key neurotrophic factors, such as BDNF [488].

##### Regeneration by IONPs

Even without cells, IONP can be applied to regenerate spinal cord injuries, particularly when combined with directed magnetic fields or/and used as vehicles to deliver therapeutic agents across the blood-brain-barrier (BBB) to the site of injury [489]. However, the optimal timing seems to be of crucial importance for a successful therapy. Jeffery et al. examined the uptake of intravenously administered IONPs into areas of experimental rodent spinal cord injury [490]. Their data suggest a “therapeutic window” during post-injury BBB impairment during which IONP-based delivery of biomolecules, particularly in combination with magnetic targeting strategies, may be most successful.

Other studies exploited the magnetic properties of the particles. For instance, Kim et al. investigated the therapeutic efficacy of exosome-mimetic and IONP-containing nanovesicles from IONP-treated human MSCs in a mouse model of spinal cord injury [491]. The exosomes were magnetically enriched in the injured spinal cord after systemic injection and were able to deliver larger amounts of therapeutic growth factors induced by the IONPs in the hMSCs to the target cells. The vesicles improved blood vessel formation, attenuated apoptosis and inflammatory responses and consequently improved spinal cord function.

Due to the influence of external magnetic fields and transactivation transduction protein (TAT), which has a remarkable membrane translocation ability, TAT-conjugated PEGlated magnetic polymeric liposomes accumulated significantly around the site of SCI and even within neurons [492].

In another study, Song et al. showed that the combination of magnetic field-mediated gene transfer and TAT-assisted intracellular delivery provides an improvement in transfection efficiency in rat spinal cord after lumbar intrathecal injection and that transgene expression can even be mediated by a magnetic field in a remote region [493]. In contrast to external magnetic fields, Zhang et al. constructed a magnetic targeting system containing a C-shaped permanent magnet and a ferromagnetic needle to generate a magnetic force large enough for growth-promoting magnetic nanomaterials to remain at the target site [494].

##### Spinal Cord Regeneration by IONP-Containing Biocomposites

Other tissue regeneration strategies include biocomposites consisting of IONP and other biocompatible materials such as hydrogels. For instance, Bhattacharyya et al. prepared a gelatin–genipin hydrogel system impregnated with IONPs that was injected into rats in a spinal cord injury model [495]. After repeated magnetic field exposure, there was a clear improvement in neuronal repair and regeneration, as evidenced by significant improvement in behavioral, electrophysiological, and morphological parameters, among others. Rose et al. developed injectable magnetoceptive, anisometric microgels that allowed alignment by external magnetic fields, resulting in parallel nerve and fibroblast growth over relatively long distances [496]. In another study, an increased sprouting of mature neurons and axons and more myelinated fibers appeared after spinal cord transection in Wistar rats and implantation of IONP-loaded agarose gels and repeated magnetic field exposure [497,498]. Chen et al. prepared temperature-dependent magnetite/polymer nanoparticles from IONPs and poly(ethylene imine)-modified poly(ethylene oxide)-poly(propylene oxide)-poly(ethylene oxide) (PEO-PPO-PEO) block copolymer. In primary mouse experiments, drug-entrapped IONPs demonstrated good biocompatibility and effective therapy for spinal cord injury [499]. Finally, Min et al. developed a magnetic field-driven graphene oxide and IONP-based hybrid pattern that enabled stable and controlled neuronal cell growth and specific cell patterning [500].

Some strategies also rely on biocomposites that have been pre-loaded with cells. In an in vitro work, a structured neuronal microphysiological system was fabricated by bio-printing IONP-loaded spheroids and PEG-based hydrogels to mimic the hierarchical structure of the nervous system [501]. The study by Syková et al. demonstrated rapid integration of IONP-loaded MSC-seeded hydrogels, based on derivatives of 2-hydroxyethyl methacrylate (HEMA) and 2-hydroxypropyl methacrylamide (HPMA), into hemisected rat spinal cords, demonstrating their potential suitability for bridging voids after spinal cord injury [457]. Finally, Adams et al. presented a potential method in which IONP-labeled canine olfactory mucosal cells (OMCs) were encapsulated in collagen hydrogels to increase the low viability of graft cells for implantation at injured sites, particularly the spinal cord, to improve regeneration outcomes [502].

#### 5.2.3. Brain

##### Monitoring by IONP-Labelled Cells

Stem cells are promising candidates for the treatment of stroke, multiple sclerosis, Huntington’s disease, Parkinson’s disease, Alzheimer’s disease, amyotrophic lateral sclerosis and epilepsy, among others [383,503,504,505]. As with all cell-based therapies, the application of IONP-labeled cells, are helpful to monitor safety, delivery, fate and therapeutic potential of the applied cells for regeneration of injured brain tissue [506,507,508,509,510,511,512,513,514,515,516,517,518,519,520,521].

##### Enhanced Brain Regeneration by IONP-Labelled Cells

IONP-labelled cells have been shown to crucially enhance the regeneration of brain tissue. Jenkins et al. showed clear differences in magnetic particle uptake and intracellular processing between neuronal subtypes [522]. They also demonstrated that oligodendrocyte precursors can be transfected efficiently and with high viability with reporter and therapeutic genes using IONPs and applied static or oscillating magnetic fields [523]. The transfected cells showed good migration, proliferation and integration ability in three-dimensional tissue engineering models using rat brain slices. Another study from Huang et al. showed that IONPs could not only label MSCs and track the fate of the cells by MRI, but also revealed that various IONPs could actively enhance the expression of the chemokine receptor CXCR4 of MSCs and improve homing efficiency in traumatic brain injury and glioblastoma mouse models compared with unlabeled MSCs [524]. Further work confirmed the therapeutic efficiency of particle-loaded MSCs. For example, loading with dextran-coated IONPs led to improved efficacy of MSCs in a mouse model of PD, including enhancement of the cell migration to the site of lesioned dopaminergic (DA) neurons and differentiation of MSCs to DA-like neurons [525].

##### IONP-Based Magnetic Cell Targeting

The intrinsic capability of IONPs enable magnetic guided cell targeting and manipulation. Schöneborn et al. and Raudzus et al. functionalized IONPs with RAS or SOS proteins, which are involved in the regulation of axonal growth as a strategy for the treatment of Parkinson’s disease (PD) that is accompanied by loss or dysfunction of dopaminergic neurons in the substantia nigra (SN). The magnetic translocation of the functionalized IONPs into the cytoplasm and from the cytoplasm to the neurite tip of human dopaminergic neurons led to the accumulation of endogenous RAS protein and the elongation of the growth cone elongated in the desired direction [526,527]. A promising approach to treat neurological diseases, including Alzheimer’s disease, is the use of MSCs. It has been shown that after intravenous injection of IONP-loaded human MSCs derived from Wharton’s Jelly (WJ-MSCs) can be magnetically directed through a Halbach magnet array into the hippocampal area in the brain of AD rats, where they improve memory and cognitive performance [528].

To treat and alleviate intracerebral hemorrhage, a rat model underwent intravenous injection of human ESC-derived spherical neural masses loaded with magnetosome-like ferrimagnetic iron oxide nanocubes. In the group with an external magnet attached to the helmet, there was significant downregulation of proinflammatory cytokines, decreased recruitment of macrophages and neutrophils, and improved neurological function [529].

##### Brain Regeneration by Application of Functionalized IONP

IONPs are frequently used as theranostic drug carriers. However, their potential neurotoxicity to the brain has not been well studied and reports show that IONPs may well have negative effects on neuronal viability. For example, Liu et al. showed dose-dependent cytotoxicity on neuronal cells and reduction in motor coordination and spatial memory in mice [530]. Despite those findings, NPs can also contribute directly to neuroprotection and neuroregeneration through specific functionalization with therapeutics and localized delivery to the injured brain [531,532]. One important substance is erythropoietin, which is used for injuries of the central nervous system and could be used in magnetic nanocarriers to enable rapid targeted delivery within the therapeutic time window by external magnetic navigation [533,534]. Another study showed that curcumin-functionalized IONPs had a strong antineurotoxic activity in the cerebellum of schizophrenic rats [535].

##### Brain Regeneration by IONP-Containing Biocomposites

Treatment of aneurysms often leads to thrombus formation and rupture near the arterial walls. In one work, revascularization of a stent was improved by coating with an elastic, magnetic and biocompatible biopolymer hydrogel based on bacterial nanocellulose (BNC) and IONPs [536]. A similar study was shown by Echeverry-Rendon et al. by fabrication of a PEG-coated magnetic BMC hydrogel membrane and IONPs for brain aneurysm treatments to render a local region of a stent scaffold magnetic and biomimetic [537]. IONP-loaded human aortic SMCs could thus be attracted to the neck region of the “aneurysms” by using a magnet to settle and proliferate.

## 6. Other Soft Tissue Regeneration and Engineering

**Figure 7 nanomaterials-11-02337-f007:**
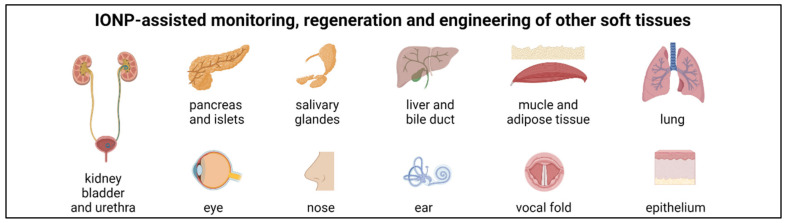
Possible targets for IONP-assisted tissue engineering and regeneration of various soft tissues (Created with BioRender.com).

The use of IONPs is considered a promising strategy tor functional restoration of different soft tissues and organs, beyond the heart and the brain. The possible targets of IONP-assisted tissue engineering and regeneration approaches reported in literature are shown in Figure 7.

### 6.1. Ear, Eye, Nose, Vocal Fold and Salivary Glands

#### 6.1.1. Ear

Stem cell transplantation is considered to be a promising strategy tor functional restoration of inner ear damage, such as sensorineural hearing loss. Thereby, it would be of greatest advantage if the course of therapy can be reliably tracked and monitored. Watada et al. showed that MSCs labeled with IONPs could be reliable monitored by MRI after transplantation into the cochleae of living animals [538]. Another study showed that MNP can penetrate the oval and round window and produce a T2 contrast effect in the inner ear, making them suitable for diagnostic purposes and potentially also as transport vehicles for therapeutic agents to treat ear damage [539]. No adverse effect on hearing was observed after intratympanic injection of ferrocene-loaded nanocarriers into guinea pigs, indicating the possibility of future applications in inner ear theranostics [540]. Magnetic guidance of IONP-labeled stem cells could increase the number of cells in the target area, and thus most likely improve the therapeutic effect in the treatment of ear damage, e.g., after exposure to ototoxic substances [541].

#### 6.1.2. Eye

Stem cell therapies are since long being explored for treatment of degenerative eye diseases [542,543]. Meanwhile, there are also approaches to treat eye diseases with the assistance of IONP. Stem cells could also serve as a therapy for glaucoma, one of the main causes of blindness. In glaucoma, the trabecular meshwork (TM) which primarily regulates intraocular pressure, has reduced cell numbers. In the ex vivo study of Snider et al. it was shown that MSCs loaded with IONPs can be targeted to the TM by external magnetic fields after injection into the anterior chamber of the eye [544]. Damage to the corneal endothelium can lead to corneal edema, opacification, and ultimately vision loss. In a feasibility study, corneal endothelial cells (CECs) were shown to be minimally affected by IONP labeling, and initial magnetic exposure even had a significant positive effect on cell viability [545]. The results suggest a potential treatment of ocular injury by IONP-loaded CECs. Ocular diseases or injuries, such as choroidal neovascularization (CNV), could also be treated by tissue engineering techniques. In the study by Ito et al., magnetic force was used to create multilayered cell sheets from magnetite liposome-loaded retinal pigment epithelium cells, which might be useful for CNV treatment [546].

#### 6.1.3. Nose

Intranasal delivery and magnetically controlled enrichment of IONP-loaded MSCs could also be used to treat olfactory damage. In an olfactory damaged mouse model, enhanced migration of magnetized MSCs could be achieved using external magnets. [547].

#### 6.1.4. Vocal Fold

The treatment of voice defects, e.g., vocal fold damage, is a strong challenge in regenerative medicine. So far, there are only a few surgical or tissue engineered approaches for the restoration of such defects [548,549,550]. Meanwhile, efforts are ongoing to build the vocal fold constructs using IONP-loaded cells. Dürr et al. isolated vocal fold fibroblasts from rabbit laryngeal heads and achieved their magnetization by incorporating IONPs [551]. The same group characterized the cellular effects of IONP uptake and demonstrated feasibility for magnetic cell guidance and possible generation of magnetic tissue-engineered 3D vocal fold constructs [552,553].

#### 6.1.5. Salivary Glands

The loss or hypofunction of salivary secretory epithelial cells, e.g., due to autoimmune diseases or radiation therapy for the treatment of cancer, could be compensated by stem cell or tissue engineering techniques. In a mouse model, the function of damaged salivary glands could be restored by cell therapy with ultra-small IONP-labeled BM-MSCs and acinar-like cells, however, the acinar-like cells showed better therapeutic potential than the BM-MSCs [554]. In another study, salivary secreting organoids/mini-glands were used to assemble IONP-tagged primary salivary gland-derived cells by magnetic levitation, which exhibited various salivary gland-specific cellular compartments and secretory function upon cholinergic stimulation [555]. Meanwhile, there is a possibility to generate salivary gland-like epithelial organoids from NP-labeled stem cells by magnetic bioprinting [556,557].

### 6.2. Kidney

Chronic kidney disease and acute kidney injury leads to high mortality rate. Current treatment options are limited to dialysis and kidney transplantation. However, tissue engineering and regenerative medicine could help reduce the problems encountered during treatment, such as graft failure, shortage of donor organs, and numerous other complications [558]. In addition to the utilization of promising cell-based approaches to restore normal kidney functions, renal tissue engineering, despite its complex structure and function, is also a potential future possibility [559].

The use of nanomaterials in nephrology has not yet been widely explored, but may be beneficial for diagnosing renal function, monitoring the course of chronic kidney disease, and producing renal drugs nanotherapies [560,561]. In an experimental rat model of mesangiolysis, Hauger et al. investigated renal glomerular homing of intravenously injected IONP-labeled MSCs and demonstrated that these cells specifically target to focal areas of glomerular damage [562]. In another study, induced pluripotent stem cell (iPSC)-derived MSCs were labeled with IONPs and injected intravenously into rats after 5/6 nephrectomy [563]. MRI studies showed that iPS-MSCs were targeted to the parenchyma of chronic kidney disease (CDK) animals and effectively protected the kidney against CKD injury.

### 6.3. Liver and Bile Duct

In the past, the overall mortality of chronic liver disease has steadily increased, causing a significant health and economic burden [564]. The most effective treatment for end-stage liver fibrosis is liver transplantation, but this is limited by shortage of organ donors and immunological rejection. A possible alternative is stem cell therapy, and potential applications including IONPs are steadily increasing [565].

Although there is evidence that long-term observation of IONP-loaded cells is not reliable, several studies have shown that IONP-labeled cells allows safe monitoring during liver therapy [566]. For instance, BM-MSCs have been demonstrated to decrease liver fibrosis and associated early liver dysplasia as well as accelerate liver healing after hepatectomy [567,568]. In another study, IONP labeling and overexpression of human hepatocyte growth factor (HGF) into MSCs improved the localization of MSCs and supported the liver repair in a rat model of liver fibrosis [569].

Functionalized IONPs have also been shown to be advantageous for liver regeneration. Fibroblast growth factor 2 (FGF2) is known to possess antifibrotic effects and promote tissue regeneration in fibrotic diseases. Eftekhari et al. found that FGF2-IONPs improved early liver fibrogenesis in vivo in the acute carbon tetrachloride-induced liver injury mouse model in contrast to free FGF2 [570].

One of the first approaches to generate in vivo-like 3D liver tissue using IONPs was in 2004 when Ito et al. used human aortic ECs loaded with cationic magnetite liposomes to form a magnetic-based accumulation of ECs on hepatocyte monolayers and thus a heterotypic, layered construct [571]. This “magnetic force-based tissue engineering” (Mag-TE), as termed by the authors, showed significantly increased albumin secretion and increased adsorption of heterotypic cells compared with single cell cultures or co-cultures without magnet.

Repair and replacement of diseased bile ducts is another area of regenerative medicine. Li et al. described a method to create biocompatible artificial bile ducts by tissue engineering [572]. A tubular composite scaffold with good mechanical properties made of PCL by 3D printing was coated externally with gelatin methacryloyl (GelMA) hydrogel with dispersed ultra-small IONPs to increase biocompatibility and allow monitoring by MRI. By co-culture with BM-MSCs, the scaffold could be almost completely colonized and might be used as an artificial bile duct for implantation in the body.

### 6.4. Islets and Pancreas

Diabetes mellitus is a serious disease, characterized by abnormally elevated blood glucose levels due to a defect in insulin production or a reduction in insulin sensitivity and function [573]. A promising strategy to restore insulin secretion in diabetes mellitus is the transplantation of pancreatic islets. In addition, the course of therapy and the localization of the grafts can be monitored by using cells that have been pre-loaded with IONPs [574,575,576,577,578,579,580].

Li et al. demonstrated the therapeutic efficacy of magnetically enhanced targeting of umbilical cord–Wharton’s jelly-derived MSCs loaded with polydopamine-coated IONPs in a clinically relevant rat model of streptozotocin-induced diabetes [581]. Magnetic targeting increased long-term cell retention in pancreatic tissue and improved islet function compared with injection of Wharton’s jelly-derived MSCs alone.

Espona-Noguera et al. followed a different approach in which IONP-loaded INS1E pseudoislets were encapsulated in alginate and alginate-poly-L-lysine-alginate (APA) microcapsules [582]. Prior to transplantation, removal of empty microcapsules by a microfluidic magnetic sorting device was performed to reduce transplant volume. After subcutaneous implantation of APA microcapsules, which have higher mechanical integrity and stability compared with alginate microcapsules, into induced diabetic Wistar rats, the animals were returned to normoglycemia for almost 17 weeks. Similarly, Delcassian et al. used IONP-loaded alginate hydrogel microcapsules containing viable islets [583]. After transplantation into immunocompetent diabetic mice, normal glycemia is restored for at least 6 weeks. IONP loading not only allowed visualization of the graft, but also allowed rapid removal of up to 94% of the transplant via a magnetically assisted retrieval device, which is important in the event of graft failure.

Other studies investigated ways to reduce the loss of transplanted cells. Loading islet cells with IONPs functionalized with siRNA against caspase-3 decreased caspase-3 expression and cell apoptosis in pancreatic islets which were transplanted into the left kidney capsule of NOD-SCID mice or infused into diabetic baboons, thereby increasing the survival of the transplanted cells [584,585].

Due to the instant blood-mediated inflammatory responses (IBMIR), engraftment of a majority of the transplanted cells fails. By using anticoagulant heparin-conjugated IONPs, IBMIR could be attenuated and stable visualization of implanted islet cells could be obtained for more than 150 days without reduction of the MRI signal [586]. Pancreatic infiltrating innate immune cells, such as macrophages, are known to play an important role in the development of acute pancreatitis [587]. Administration of IONP clodronate-containing liposomes attenuated the pathological changes in the pancreas and kidneys of rats with acute pancreatitis [588].

### 6.5. Bladder and Urethra

Cell therapy is also a promising method for the treatment of diseased or injured bladder or ureter, which can be monitored noninvasively by using IONPs. Lee et al. demonstrated that transplantation of magnetic NP-labeled B10 human MSCs into the bladder wall of rats with spinal cord injury inhibits bladder fibrosis and ameliorates bladder dysfunction [589] and Kim et al. showed that after periurethral injection of IONP-labeled human amniotic fluid stem cells (hAFSCs) can restore urethral sphincter competence in a mouse model of stress urinary incontinence (SUI) [590]. Another study using a rat model of bladder outlet obstruction, transplantation of IONP-loaded MSCs inhibited bladder fibrosis and mediated recovery of bladder dysfunction [591]. In a rabbit model of urogenital tuberculosis (TB), which often results in bladder contraction, a reduction in urinary reservoir capacity and eventually true microcystitis to complete obliteration, interstitial injection of IONP-loaded MSCs in combination with standard anti-TB treatment was shown to restore bladder function [592]. One strategy to treat the insufficiency of the striated urethral sphincter is the use of myogenic progenitor cells. To localize myofiber implants, Rivière et al. magnetically labeled the implants, allowing monitoring for up to 1 month in a female pig model [593].

The use of external magnetic fields can significantly enhance the regenerative effect of IONP-loaded cells. A study by Sadahide et al. demonstrated improved repair of resected bladder tissue in rabbit models with damaged bladder by injection of IONPs-loaded MSCs and the presence of a directed external magnetic force than without a magnetic field or when using unlabeled MSCs [594]. A potential therapeutic method for the treatment of SUI was developed by Wang et al. [595]. Targeted magnetic accumulation of IONP-labeled ASCs improved the retention rate of transplanted cells and restored sphincter structure and function in a rat SUI model.

Biocomposites may also be utilized for tissue engineering of bladder and urethral tissues. In one study, a minimally invasive approach was developed to treat SUI using an injectable bulking agent composed of ECM fragments of ASC sheets [596]. The fragments fully integrated into the surrounding tissue within 1 week, and after 4 weeks, host cells had regenerated around the fragments. In addition, new smooth muscle tissue had formed around the fragments. A potential approach for bladder tissue engineering was described in the study by Yudintceva et al. [597]. Herein, a bilayer scaffold of PLA/silk fibroin was generated and seeded with IONP-labeled and thus MRI-traceable allogeneic BM-MSCs. After transplantation into a rabbit model of partial bladder wall cystectomy, biointegration of the scaffold seeded with allogeneic BMSCs was shown to improve bladder tissue regeneration and function compared with unseeded scaffolds. In another study, the same group used bilayer PLA/PCL scaffolds seeded with IONP-loaded MSC to reconstruct the urethra in a rabbit model. The scaffolds integrated with the surrounding urethra tissue, and exhibited less fibrosis and inflammatory cell infiltration than conventional urethroplasty using an autologous buccal mucosa graft [598]. In another work, a three-dimensional bladder patch was developed that was composed of a porous polyglycolic acid scaffold and multilayers of ultra-small IONP-labeled ASC [599]. The bladder patches could be monitored by MRI in a rat model, and the enhancement of urothelial, smooth muscle, neuronal and blood vessel regeneration was confirmed by immunofluorescence analysis. Further urodynamic tests showed that bladder function could be restored with increased capacity. Ultra-small IONP-labeled bladder patches thus represent a promising image-guided therapeutic strategy for bladder regeneration. A study by Zhou et al. demonstrated the construction of a tissue engineered bionic urethra using cell layer technology [600]. After isolating stem cells from adipose tissue, epithelial cells of the oral mucosa and fibroblasts of the oral mucosa and labeling them with IONPs, they first produced individual cell layers. Hierarchically tubularized, three-layered urethras were generated by wrapping a silicone tube in an orderly fashion. After subcutaneous grafting to promote revascularization and biomechanical strength, a small area of the penile urethra was replaced with the tissue-engineered urethras in the canine model. At 3 months after transplantation, MRI still showed the three-layered structure. In addition, the density of the blood vessels was almost restored. The study shows that functional tissue engineering of structurally complicated structures such as urethras is possible. Finally, Ito et al. fabricated a scaffold-free cell tube by creating a cell sheet from IONP-loaded urothelial cells. After rolling a cylindrical magnet onto the cell sheet and magnetically attracting the cells, a tube was formed from which the magnet was subsequently removed [601].

### 6.6. Tissue Glue, Wound Regeneration and Skin Engineering

The main causes of the penetration of dangerous pathogens, bleeding or postoperative wound complications are injuries or insufficient wound healing of the barrier function of affected surfaces, tissues or organs.

Tissue adhesives represent one possibility for non-invasive wound closure [602]. In the course of the last decades, a large number of different natural, synthetic, semisynthetic and biomimetic pressure-sensitive adhesives have been developed [603]. Further improvements in product properties could be made possible by the use of nanotechnology, for instance through the principle of particle nanobridging, i.e., adhesion through aqueous nanoparticle solutions [604]. Through nanobridging, Meddahi-Pellé achieved closure of deep wounds in rat skin and liver using Stöber silica and iron oxide nanoparticles [604]. To date, there are several other studies investigating different tissue adhesives and wound dressings for wound healing and restoration of barrier integrity [605,606]. Matter et al. prepared bioactive tissue adhesives with different nanoparticles by flame spray pyrolysis [607]. The biocompatibility and adhesive properties of six different metal oxide particles were tested in an ex vivo porcine small intestinal lap joint model. In particular, the ceramic bioglass greatly promoted coagulation and exhibited remarkable adhesive properties. Wang et al. prepared a magnetic adhesive from steel microparticles and a cyanoacrylate adhesive (Loctite 4014) [608]. Intraluminal injection of the magnetic adhesive into ex vivo porcine colonic segments and a magnet at the injection site resulted in polymerization of an intraluminal, mucosally adherent coagulum that allows adequate retraction of the bowel with little tissue trauma during minimally invasive surgery. Another study demonstrated the preparation of a composite thin film of PCL-IONPs, which exhibited both antimicrobial properties and good cytocompatibility with NIH 3T3 mouse fibroblasts [609].

Grumezescu et al. demonstrated the development, characterization and evaluation of an absorbable and antimicrobial wound dressing based on anionic polymers (sodium alginate, carboxymethylcellulose) and Fe₃O₄ nanoparticles loaded with usnic acid [610]. The results showed low cytotoxicity to human progenitor cells with good antimicrobial activity, suggesting potential applications in tissue regeneration. Bunea et al. developed a promising wound dressing based on a silk fibroin-IONP scaffold that exhibited good cytocompatibility on human ASCs [611]. Anghel et al. modified a textile wound dressing by coating with a nanofluid containing IONPs and natural microbicidal compounds, such as vegetal eugenol and limonene [612]. The hybrid phyto-nanostructured coating exhibited significant anti-adherence and anti-biofilm properties against two of the most important bacterial pathogens implicated in wound infections, *P. aeruginosa* and *S. aureus*.

The magnetic properties of IONPs can also be exploited to achieve accelerated wound healing. Accelerated wound repair using IONPs functionalized with fibroblast growth factor bFGF and an external magnetic field (eMF) was shown by Wu et al. [613] The particles exhibited sustained release of bFGF, increased cell proliferation and promoted macrophage polarization toward an anti-inflammatory (pro-healing) M2 phenotype especially in presence of eMF. In a rat model of laser-induced skin injury, administration of IONP-loaded MSCs and their magnetically enhanced migration to the injury site improved skin regeneration and enhanced anti-inflammatory effects and angiogenesis, compared with MSC injection alone [614]. Improved wound healing through accelerated wound closure, reduced scar width, and enhanced angiogenesis was also achieved with exosomes derived from BMSCs preconditioned with IONPs and a static magnetic field compared with IONPs-free exosomes in a rat wound healing model [615]. Heun et al. have developed a novel wound healing strategy for pathophysiological conditions with impaired wound healing using a site-specific gene transfer technique based on magnetic targeting of IONP-lentivirus complexes that ultimately controls hypoxia inducible factor 1α (HIF-1α)-dependent wound healing angiogenesis via modulation of the tyrosine phosphatase activity of SHP-2 [616].

In addition to a wide variety of approaches to skin regeneration, there are also efforts toward skin tissue engineering [617]. Zhang et al. fabricated three-dimensional fibrous composite membranes from the tri-block copolymer PCL-PEG-PCL with embedded IONPs [618]. In vitro cell culture with murine NIH 3T3 fibroblasts showed that the composite fibers not only exhibited low cytotoxicity, but also provided a suitable scaffold for cell adhesion and may potentially be used for skin tissue engineering. Paun et al. demonstrated a proof of concept of magnetically driven 2D cell organization on superparamagnetic micromagnets fabricated by laser direct writing by two-photon polymerization of a photopolymerizable superparamagnetic composite [619]. Under a static magnetic field, fibroblasts adhered exclusively to the micromagnets, resulting in precise 2D cell organization on the chessboard-like microarray, suggesting a potential suitability skin tissue engineering. In another in vitro study, electrospun nanofibers composed of PVA and IONPs showed good biocompatibility against human skin fibroblast cells and could potentially be used as biomaterials for tissue engineering scaffolds [620].

### 6.7. Muscle and Adipose Tissue

Skeletal muscle repair requires tissue engineering strategies that incorporate scaffolds of biomaterials and cells capable of successfully restoring physiologically relevant functions [621]. In addition, non-invasive imaging techniques help to evaluate and monitor the therapy. For example, high-resolution images of mesoangioblasts could be made indirectly by their IONP loading in skeletal and cardiac muscle in a mouse model of muscular dystrophy to monitor the progress of stem cell-mediated repair of muscle tissue [622]. Similarly, IONP-labeled ASCs could be reliably observed for up to 8 weeks after intramuscular injection into the ischemic leg in a mouse model of hind limb ischemia [623].

The in vivo distribution and migration of MSCs containing very small IONPs could be reliably observed by MRI after transplantation into the soleus muscle of rat model of open crush injury [624]. In another work on muscle tissue regeneration, it was shown that intra-arterially injected stem cells migrate specifically into the damaged muscle tissue after several rounds of recirculation [625]. Current treatments for anal incontinence, a condition that affects 11–15% of the adult population, are often ineffective. Potential therapies for the treatment of anal sphincter injuries is cellular therapy with stem cells and progenitor cells [626]. Labeling of muscle progenitor cells with ultra-small IONPs allowed serial MRI monitoring of transplanted cells in an experimental rabbit model of anal sphincter repair [627].

Fabrication of well-organized structures remains a challenge in tissue engineering. In the study by Lee et al., a three-dimensional (3D) cell-dense tissue was fabricated from fiber bundles consisting of composite fibers of PLGA/IONPs using an electrospinning technique and then seeded with C2C12 myoblasts that differentiated into multinucleated myotubes [628]. An applied external magnetic field caused the cell rods to assemble into a 3D tissue with a highly ordered architecture similar to those in native skeletal muscle tissues. Another study used IONP-loaded C2C12 myoblast that differentiated into myogenin-positive multinucleated myotubes in differentiation medium to produce a very dense and oriented skeletal muscle tissue of ring-shaped multilayered cell sheets by magnetic tissue engineering [629].

In regenerative medicine, there are efforts to restore defective adipose tissue. In one work, hyaluronic-based magnetic nanospheres were prepared with covalently immobilized dexamethasone (Dex) as an adipogenic factor to induce adipogenesis for adipose regeneration [630]. The delivery of Dex could be easily controlled by external magnetic field and demonstrated a high efficiency to promote the viability of ASCs, indicating a potential usage for soft tissue engineering approaches.

One of the most common complications in spine surgery is epidural fibrosis, which results from destruction of epidural adipose tissue after laminectomy. ASC isolated from subcutaneous fat and a porous PLGA scaffold were used to form an engineered adipose tissue, which successfully led to the regeneration of epidural fat and prevented the formation of an epidural scar in a rabbit model of dorsal laminectomy [631]. In addition, the NP-labeled ASCs could be used for MRI visualization for up to four weeks.

White adipose tissue (WAT) is of great interest for tissue engineering and cell-based therapies because it is a rich source of differentiated adipocytes, stromal mesenchymal progenitors/ASCs, and ECs and infiltrating leukocytes. Tseng et al. and Daquinag et al. developed and used a three-dimensional (3D) levitation tissue culture system based on magnetic nanoparticles [632,633]. They demonstrated that 3D intercellular signaling recapitulates WAT organogenesis and relative cell positioning in organoids called adipospheres better than 2D cultures. The 3D technique could thus support potential WAT-based cell therapies in the future.

### 6.8. Lung Tissue

Stem cells have been extensively studied in the field of regenerative medicine and can be effectively delivered to the target site by magnetic targeting to increase the number of cells and their retention and improve the effect of the delivered cells [413]. Silva et al. administered 2,3-DMSA-functionalized IONP into a mouse model of silicosis via the jugular vein and used magnetic targeting with fixed magnets in the thoracic region to improve retention of the particles in the injured lung [634].

Other pulmonary diseases, such as cystic fibrosis (CF) and tuberculosis (TB), can also benefit from NP-based diagnostic and treatments [635,636,637,638]. For instance, in CF, conjugation of IONPs with appropriate antibiotics could enhance drug delivery through the mucus and biofilm to bacterial infections, which commonly occurs in CF [639]. Further studies demonstrated the possibility of magnetic targeting of aerosols, in which drugs of choice can be enriched with dose accuracy into diseased lung regions, thereby reducing undesirable side effects [640].

There are only limited reports about lung tissue engineering. In the study by Dzamukova et al., adenocarcinomic human alveolar epithelial cells (A549) and human skin fibroblasts were labeled with (poly)allyl amine-coated IONPs [641]. A magnetic force enabled the formation of a two-layered scaffold-free lung-mimicking tissue, showing the characteristic porous alveoli mimicking native morphologies.

## 7. Conclusions and Discussion

In recent years, many advances in the development and use of nanomaterials for medical applications have been reported. The use of NPs is favored by their high modifiability, where the properties of the particles can be tailored exactly to the specific application by the selected manufacturing method, coating or/and functionalization. Besides the possibility of IONP-based visualization of scaffolds in the field of tissue engineering, IONPs can be adapted to any specific application where cellular effects are controlled by loading and delivery of bioactive agents. Especially in the field of cell therapy, there are countless possibilities to beneficially influence the therapeutic outcome, be it through non-invasive monitoring of IONP-labeled cells or through a positive effect of NPs on the differentiation and regeneration capacity of the administered cells. Thus, IONPs offer a wide range of potential applications.

However, there are many challenges for successful translation to the clinic [642]. Firstly, the formation and composition of the protein corona, which forms within seconds of application into the body and interacts strongly with the biological system, is not yet fully understood and may cause unexpected results in clinical application [643]. Extensive reliable toxicological studies are another essential prerequisite for successful translation into clinical applications. In particular, the interference of nanoparticles with numerous classical toxicological detection methods forces the use of alternative methods such as real-time label-free analyses, multiparameter staining for flow cytometry and fluorescence microscopy [644,645,646]. Moreover, the use of 2D cell culture is not sufficient for in vitro evaluation of particles, but should be extended with 3D cell culture and flow models before promising NPs can be evaluated in animal models [647]. The body’s immune response is an additional significant problem that can result in graft rejection. Side effects of administered immunosuppressants towards transplanted cells could be mitigated, for example, by slow release from hydrogels, immunoisolation of transplanted cells in semipermeable microcapsules or magnetically controlled local release [648,649,650]. Finally, magnetically controlled targeting of IONPs or IONP-labeled cells requires a sufficiently strong external magnetic field and IONPs with high saturation magnetization. However, most commercially available magnets only provide a reasonable high magnetic force for distances of a few millimeters, and many IONP exhibit insufficient magnetizability, which could be optimized by precisely adjusting various parameters such as size, composition size distribution, and structural and magnetic properties [26,651]. Therefore, the development of optimized synthesis methods, innovative magnetic field configurations and/or new stronger magnets is necessary to enable targeted and efficient delivery to deeper regions of the body [652,653].

Thus, to achieve translation to the clinic, the complete workflow from nanoparticle synthesis according to the current good manufacturing practice standards with accurate physicochemical characterization, to comprehensive in vitro and in vivo toxicological studies including biodistribution and efficacy studies must be performed [654]. Hence, there are only few IONPs that have successfully completed the translation to the clinics thus far. Despite this fact, the successful use of IONPs in many areas of nano-medical research raises hopes about further IONP formulations to be approved for clinical applications in a not-so-distant future.

## References

## Data Availability

Not applicable.

## Abbreviations

APA	alginate-poly-L-lysine-alginate	MMPs	matrix metalloproteases
APTS	acetylated 3-aminopropyltrimethoxysilane	MPI	magnetic particle imaging
ARCs	adipose-derived regenerative cells	MPO	myeloperoxidase
ASCs	adipose-derived stem cells	MRI	magnetic resonance imaging
BC	bacterial cellulose	MRI	magnetic resonance imaging
Bcl-2	B-cell lymphoma 2	MSCs	mesenchymal stem cells
BDNF	brain-derived neurotrophic factor	NE	nanoemulsion
BM-EPCs	bone marrow-derived endothelial progenitor cells	NGF	nerve growth factor
BMMs	biomimetic magnetic microrobots	n-HA	nano-hydroxyapatite
BMP-2	bone morphogenetic protein 2	NIRF	near-infrared fluorescence
BM-SCs	bone marrow-derived stem cells	NK	nattokinase
BNC	bacterial nanocellulose	NPs	nanoparticles
CCR2	chemokine (C-C motif) receptor 2	OECs	olfactory ensheathing cells
CDK	chronic kidney disease	OMCs	olfactory mucosal cells
CECs	corneal endothelial cells	OxLDL	oxidized low-density lipoproteins
CF	cystic fibrosis	PAA	polyacrylic acid
ChABC	chondroitinase ABC	PCL	poly-ε-caprolactone
CMs	cardiomyocytes	PEG	polyethylene glycol
CNTs	carbon nanotubes	PEI	polyethylenimine
CPC	calcium phosphate cement	PEO	polyethylene oxide
cRGD	cyclic arginine-glycine-aspartate	PET-CT	positron emission tomography–computed tomography
CSCs	cardiosphere-derived stem cells	PGA	polyglycolic acid
CTA	computed tomographic angiography	PLA	poly(l-lactide)
dCCA	decellularized porcine common carotid artery	PLGA	poly(lactic-co-glycolic acid)
Dex	dexamethasone	PMAO	poly(maleicanhydride-alt-1-octadecene)
Dexa	dexamethasone phosphate	POC	poly(1,8-octamethylene citrate)
DGR		Ptx	paclitaxel
DMSA	dimercaptosuccinic acid	PU	polyurethane
DPSCs	dental pulp stem cells	PVA	polyvinyl alcohol
DS	dextran sulfate	PVDF	polyvinylidene fluoride
ECCs	embryonic cardiac cells	PVDF	polyvinylidene difluoride
ECM	extracellular matrix	RGCs	retinal ganglion cells
ECs	endothelial cells	RGD	arginine-glycine-aspartate
EGFP-EGF1	enhanced green fluorescent protein with the first epidermal growth factor domain	ROS	reactive oxygen species
EMMPRIN	ECM metalloproteinase inducer	scFv	single-chain variable fragment antibody
eNOS	endothelial nitric oxide synthase	SCs	Schwann cells
EPCs	endothelial progenitor cells	sGAG	sulfated glycosaminoglycan
ePTFE	expanded polytetrafluoroethylene	SK	streptokinase
ESCs	embryonic stem cells	SkMB	skeletal myoblasts
FGF2	fibroblast growth factor 2	SMCs	smooth muscle cells
FLI	fluorescence imaging	SPECT	single photon emission computed tomography
GDNF	glial derived neurotrophic factor	SR-A	macrophage scavenger receptor type A
GFP	green fluorescent protein	SS	stainless steel
HA	hydroxyapatite	SUI	stress urinary incontinence
hAECs	human aortic endothelial cells	TEVGs	tissue-engineered vascular grafts
HDL	high-density lipoproteins	TGF	transforming growth factor
hESCs	human embryonic stem cells	tPA	tissue plasminogen activator
hVEGF	human vascular endothelial growth factor	UK	urokinase
IBMIR	instant blood-mediated inflammatory responses	VCAM-1	endothelial vascular adhesion molecule-1
IONPs	iron oxide nanoparticles	VEGF	vascular endothelial growth factor
LIFU	low intensity focused ultrasound irradiation	VEGFR-2	vascular endothelial growth factor receptor-2
MCP-1	monocyte chemoattractant protein-1	WAT	white adipose tissue
